# Supplementary material for: Do Vegetarian Diets Provide Adequate Nutrient Intake during Complementary Feeding? A Systematic Review
Source: Nutrients. 2022 Aug 31;14(17):3591. doi: 10.3390/nu14173591 (PMC9459879; doi:10.3390/nu14173591)

File S1. LITERATURE SEARCH AND SCREENING RESULTS

Key questions

1. Does complementary feeding completely or partially free of animal-source foods in healthy infants result in significantly different auxological development and/or growth compared with that of infants on a balanced omnivorous diet?
2. Does complementary feeding completely or partially free of animal-source foods in healthy infants/toddlers result in a psychomotor development that is significantly different from that of infants on a balanced omnivorous diet?
3. Do healthy infants on a complementary feeding that is completely or partially free of any animal-based foods

    3.1. have a different risk of developing NCDs (Obesity/Overweight/Hypertension, Diabetes Mellitus)?

    3.2. have a different risk of developing vitamin or other micronutrient deficiencies

    3.3. have a different risk of developing infections and related outcomes

    compared to infants on a balanced omnivorous diet?

PICOs

1.

P. Will a healthy infant at the beginning and during the complementary feeding period (6-24 months)
I. who is offered a diet completely or partially devoid of food of animal origin
C. towards an infant who, in the same period, is offered a balanced omnivorous diet
O. have significantly different growth and development?

2.

P. Will a healthy infant at the beginning and during the complementary feeding period (6-24 months)
I. who is offered a diet completely or partially devoid of food of animal origin
C. towards an infant who, in the same period, is offered a balanced omnivorous diet
O. have significantly different psychomotor development?

3.1.

P. Will a healthy infant at the beginning and during the complementary feeding period (6-24 months)
I. who is offered a diet completely or partially devoid of food of animal origin
C. towards an infant who, in the same period, is offered a balanced omnivorous diet
O. have a different risk of developing NCDs (Obesity / Overweight, Hypertension, Diabetes mellitus)?

3.2.

P. Will a healthy infant at the beginning and during the complementary feeding period (6-24 months)
I. who is offered a diet completely or partially devoid of food of animal origin
C. towards an infant who, in the same period, is offered a balanced omnivorous diet
O. have a different risk of developing vitamin or micronutrient deficiencies?

3.3.

P. Will a healthy infant at the beginning and during the complementary feeding period (6-24 months)
I. who is offered a diet completely or partially devoid of food of animal origin
C. towards an infant who, in the same period, is offered a balanced omnivorous diet
O. have will it have a different risk of developing infections and related outcomes?

KEYWORDS

Population

No age limit
[infant]/lim OR [child]/lim OR [preschool]/lim

'child'/exp
'infant'/exp
'infant'
'preschool child'/exp
'adolescent'/exp
'adolescent'
'teenager'

Exposure Factors

Vegetarian*
Lacto-ovo–vegetarian*
Non vegetarian*
Veg*
Dietary pattern*
Diet, Vegan
'dietary patterns'
'vegetarian diet'/exp
'vegetarian diet'
MeSH descriptor: [Diet, Vegetarian]
"Infant Nutritional Physiological Phenomena"
"complementary feeding"
"weaning"

Comparison

Diet, Western
Diet, Mediterranean
Diet
'healthy diet'/exp
'western diet'/exp
'mediterranean diet'/exp

Outcomes

"Growth and Development"[Mesh]
"Growth"[Mesh]
"Growth Charts"[Mesh]
"Body Height"[Mesh]
"Body Weight"[Mesh]
"Body-Weight Trajectory"[Mesh]
"Body Weight Changes"[Mesh]
“Overweight"[Mesh]
"Obesity"[MeSH Terms]
"Body Size"[MeSH Terms]
"Growth and Development"[MeSH Terms]
"Weight Gain"[MeSH Terms]
" growth and development"
"child neurodevelopment"
"brain development"
"cerebral development"
'growth physiology':ti,ab,kw
'body height':ti,ab,kw
'growth'/exp
'growth, development and aging'/exp
'body weight'/exp
'body mass'/exp
'brain development'/exp
'brain cortex development'
'brain development'
'cerebral development'
'development, brain'
'child development'/exp
'child development' OR 'development, child'
'infant development'
'non communicable disease'/exp
'diabetes mellitus'/exp
'hyperglycemia'/exp
hypertension'/exp
'blood pressure'/exp OR 'elevated blood pressure'/exp
'obesity'/exp

'body mass'/exp
'anemia'/exp
‘iron deficiency’
'infection'/exp
'vitamin deficiency'/exp
'vitamin deficiency'
'nutritional deficiency'/exp

Guidelines search

PubMed

#1
("vegetarian*"[All Fields] OR "lacto ovo vegetarian*"[All Fields] OR ("Non"[All Fields] AND "vegetarian*"[All Fields]) OR "veg"[All Fields] OR (("diet"[MeSH Terms] OR "diet"[All Fields] OR "dietary"[All Fields] OR "dietaries"[All Fields]) AND "pattern*"[All Fields]) OR ("diet, vegetarian"[MeSH Terms] OR ("diet"[All Fields] AND "vegetarian"[All Fields]) OR "vegetarian diet"[All Fields] OR ("diet"[All Fields] AND "vegetarian"[All Fields]) OR "diet vegetarian"[All Fields]) OR ("diet, vegan"[MeSH Terms] OR ("diet"[All Fields] AND "vegan"[All Fields]) OR "vegan diet"[All Fields] OR ("diet"[All Fields] AND "vegan"[All Fields]) OR "diet vegan"[All Fields]) OR "diet, vegan"[MeSH Terms] OR "diet"[All Fields] OR "vegan"[All Fields] OR "vegan diet"[All Fields] OR "vegan"[All Fields] OR "diets"[All Fields] OR "vegan diets"[All Fields]) AND ((y_5[Filter]) AND (consensusdevelopmentconference[Filter] OR consensusdevelopmentconferencenih[Filter] OR guideline[Filter] OR practiceguideline[Filter]))

EMBASE

#1

('vegetarian diet'/exp OR 'vegetarian diet') AND ('practice guideline'/exp OR 'practice guideline') AND [2017-2022]/py

UPTODATE https://www.uptodate.com/home

vegetarian diet

Society Guideline Links: “*Healthy diet in children and adolescents*"

SOCIETY GUIDELINE LINKS:

Indian Academy of Pediatrics (IAP) https://iapindia.org/adhd-iap-national-guidelines/

National Health and Medical Research Council (NHMRC): Australian dietary guidelines

World Health Organization (WHO): https://www.who.int/publications/i/item/9789241515856

Health Canada: https://food-guide.canada.ca/en/

Canadian Paediatric Society (CPS): https://cps.ca/en/documents/position/nutrition-healthy-term-infants-6-to-24-months

Centers for Disease Control and Prevention (CDC): https://www.cdc.gov/nutrition/strategies-guidelines/index.html

US Department of Agriculture (USDA) and US Department of Health and Human Services (HHS): https://www.dietaryguidelines.gov/resources/2020-2025-dietary-guidelines-online-materials

https://www.dietaryguidelines.gov/resources/about-process-2020

Public Health England (PHE): https://www.gov.uk/government/publications/the-eatwell-guide

American Academy of Pediatrics (AAP) https://www.aap.org/en-us/Pages/Default.aspx

EPA/UNEPSA http://www.epa-unepsa.org/

Guidelines International Network https://g-i-n.net/

U.S. Preventive Services Task Force https://www.uspreventiveservicestaskforce.org/uspstf/

ESPGHAN https://www.espghan.org/home

Sistema Nazionale Linee Guida https://www.iss.it/linee-guida1

Società Italiana di Pediatria (SIP) http://www-sip.it/

Società Italiana di Pediatria Preventiva e Sociale (SIPPS) https://www.sipps.it/

Società Italiana di Nutrizione Pediatrica (SINUPE) https://sip.it/2017/09/21/sinupe-societa-italiana-di-nutrizione-pediatrica/

Figure S1a: Guidelines search flow diagram

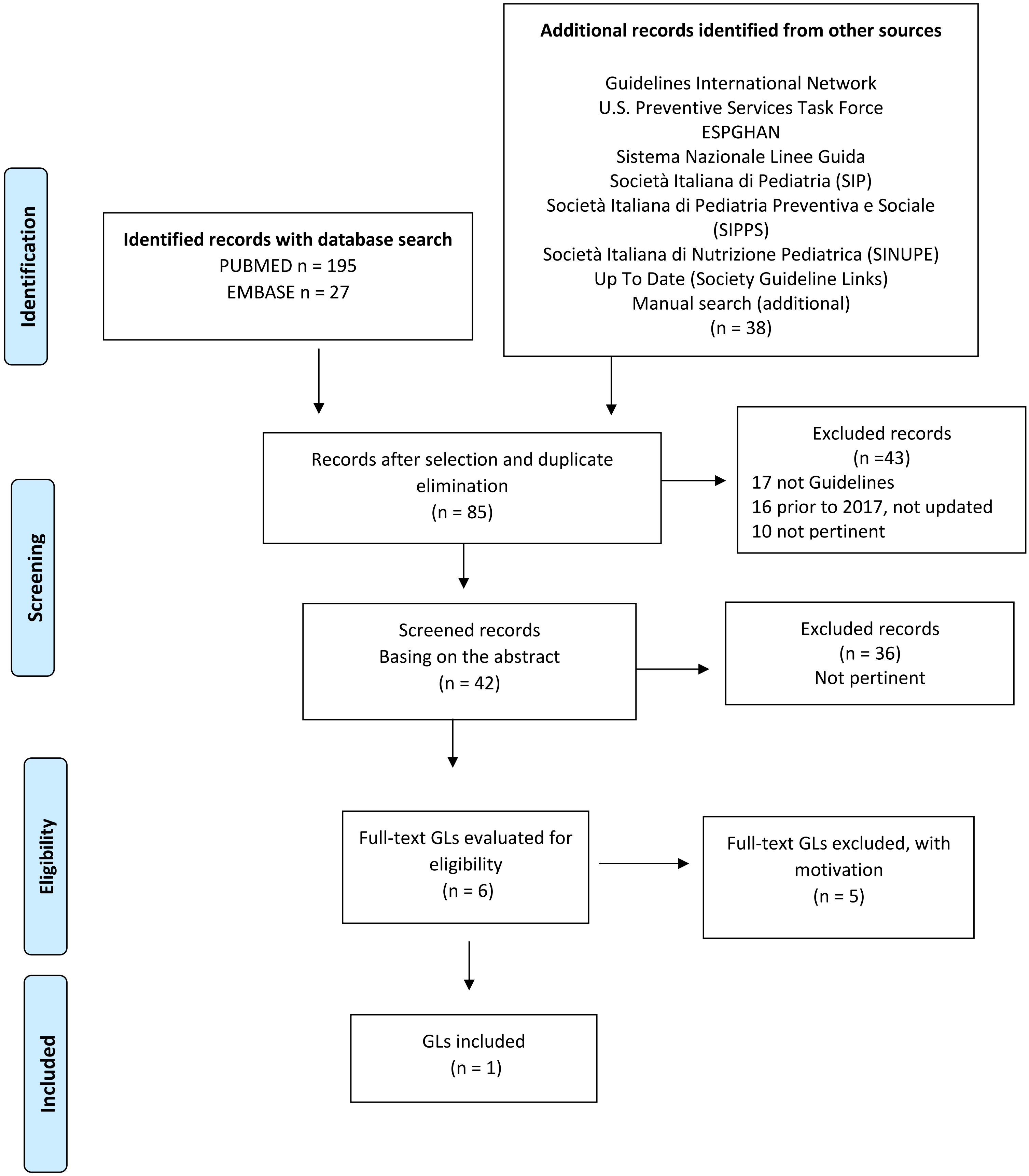

Key question

1. Does complementary feeding completely or partially free of animal-source foods in healthy infants result in significantly different auxological development and/or growth compared with that of infants on a balanced omnivorous diet?

P.I.C.O.

P. Will a healthy infant at the beginning and during the complementary feeding period (6-24 months)
I. who is offered a diet completely or partially devoid of food of animal origin
C. towards an infant who, in the same period, is offered a balanced omnivorous diet
O. have significantly different growth and development?

Systematic Reviews search

COCHRANE LIBRARY

in Title Abstract Keyword (Word variations have been searched)

"Infant Nutritional Physiological Phenomena"
"complementary feeding"
" weaning"
“growth”
" growth and development"
“overweight”
“child health”
"child neurodevelopment"
"brain development"
"cerebral development"
“Diet, Vegetarian”

Custom date range Topics: **01.01.2012 - 03.31.2022**

PubMed

#1
(("diet, mediterranean"[MeSH Terms] OR "diet, healthy"[MeSH Terms] OR "diet, vegetarian"[MeSH Terms] OR "diet, western"[MeSH Terms] OR "dietary pattern*"[All Fields] OR "veg"[All Fields]) AND ("Overweight"[MeSH Terms] OR "Obesity"[MeSH Terms] OR "Body Size"[MeSH Terms] OR "Growth and Development"[MeSH Terms] OR "Growth"[MeSH Terms] OR "Growth Charts"[MeSH Terms] OR "Weight Gain"[MeSH Terms])) AND **((y_10[Filter**]) AND (meta-analysis[Filter] OR systematicreview[Filter]) AND (allchild[Filter]))

EMBASE

#1
('healthy diet'/exp OR 'western diet'/exp OR 'mediterranean diet'/exp OR 'dietary patterns' OR 'vegetarian diet'/exp) AND ([systematic review]/lim OR [meta analysis]/lim) AND ([infant]/lim OR [child]/lim OR [adolescent]/lim) AND [**2012-2022**]/py

Studies search

Cochrane

#1
("Infant Nutritional Physiological Phenomena" OR "complementary feeding" OR " weaning") AND (“growth” OR " growth and development" OR “overweight” OR “child health” OR "child neurodevelopment" OR "brain development" OR "cerebral development" OR “Diet, Vegetarian”)
in Title Abstract Keyword - with Cochrane Library publication date Between Jul 2016 and Jun 2022, in Trials (Word variations have been searched)

PubMed

#1

(("diet, mediterranean"[MeSH Terms] OR "diet, healthy"[MeSH Terms] OR "diet, vegetarian"[MeSH Terms] OR "diet, western"[MeSH Terms] OR "dietary pattern*"[All Fields] OR "veg"[All Fields]) AND ((((("Growth and Development"[Mesh]) OR "Growth"[Mesh]) OR "Growth Charts"[Mesh]) OR "Body Size"[Mesh] OR "Obesity"[MeSH Terms] OR "Pediatric Obesity"[MeSH Terms] OR "Overweight"[MeSH Terms] OR "Body Mass Index"[MeSH Terms] OR "Body Weight Changes"[MeSH Terms] OR "Body Weight"[MeSH Terms] OR "Body Composition"[MeSH Terms] OR "Nutritional Status"[MeSH Terms])) AND ((casereports[Filter] OR clinicalstudy[Filter] OR clinicaltrial[Filter] OR clinicaltrialphaseiv[Filter] OR comparativestudy[Filter] OR controlledclinicaltrial[Filter] OR multicenterstudy[Filter] OR observationalstudy[Filter] OR pragmaticclinicaltrial[Filter] OR randomizedcontrolledtrial[Filter]) AND (2016/7/1:2022/3/31[pdat]) AND (allchild[Filter]))

# 2

(("body size"[Title/Abstract] OR "body size"[MeSH Terms] OR "obesity"[Title/Abstract] OR "obese"[Title/Abstract] OR "overweight"[MeSH Terms] OR "obesity"[MeSH Terms] OR "overweight"[Title/Abstract] OR "adipos*"[Title/Abstract] OR "adiposity"[MeSH Terms] OR "body composition"[MeSH Terms] OR "body fat distribution"[MeSH Terms] OR "body fat"[Title/Abstract] OR "body weight"[Title/Abstract] OR "body weight"[MeSH Terms] OR "birth weight*"[Title/Abstract]) AND ("diet, mediterranean"[MeSH Terms] OR "diet, healthy"[MeSH Terms] OR "diet, vegetarian"[MeSH Terms] OR "diet, western"[MeSH Terms] OR "dietary pattern*"[All Fields] OR "veg"[All Fields])) AND ((casereports[Filter] OR clinicalstudy[Filter] OR clinicaltrial[Filter] OR clinicaltrialphaseiv[Filter] OR comparativestudy[Filter] OR

controlledclinicaltrial[Filter] OR multicenterstudy[Filter] OR observationalstudy[Filter] OR pragmaticclinicaltrial[Filter] OR randomizedcontrolledtrial[Filter]) AND (2016/7/1:2022/3/31[pdat]) AND (allchild[Filter]))

EMBASE

# 1
('vegetarian diet'/exp OR 'diet, vegetarian' OR 'plant-based diet' OR 'vegetarian diet' OR 'vegetarian eating patterns' OR 'vegetarianism') AND ('weight, mass and size'/exp OR 'growth'/exp OR 'body height'/exp) AND ([infant]/lim OR [preschool]/lim OR [school]/lim OR [adolescent]/lim) AND [2016-2022]/py AND ('case report'/de OR 'case control study'/de OR 'clinical trial'/de OR 'cohort analysis'/de OR 'comparative study'/de OR 'controlled clinical trial'/de OR 'controlled study'/de OR 'cross sectional study'/de OR 'intervention study'/de OR 'longitudinal study'/de OR 'multicenter study'/de OR 'observational study'/de OR 'prospective study'/de OR 'randomized controlled trial'/de OR 'randomized controlled trial topic'/de OR 'retrospective study'/de)

Figure S1b: SRs search flow diagram

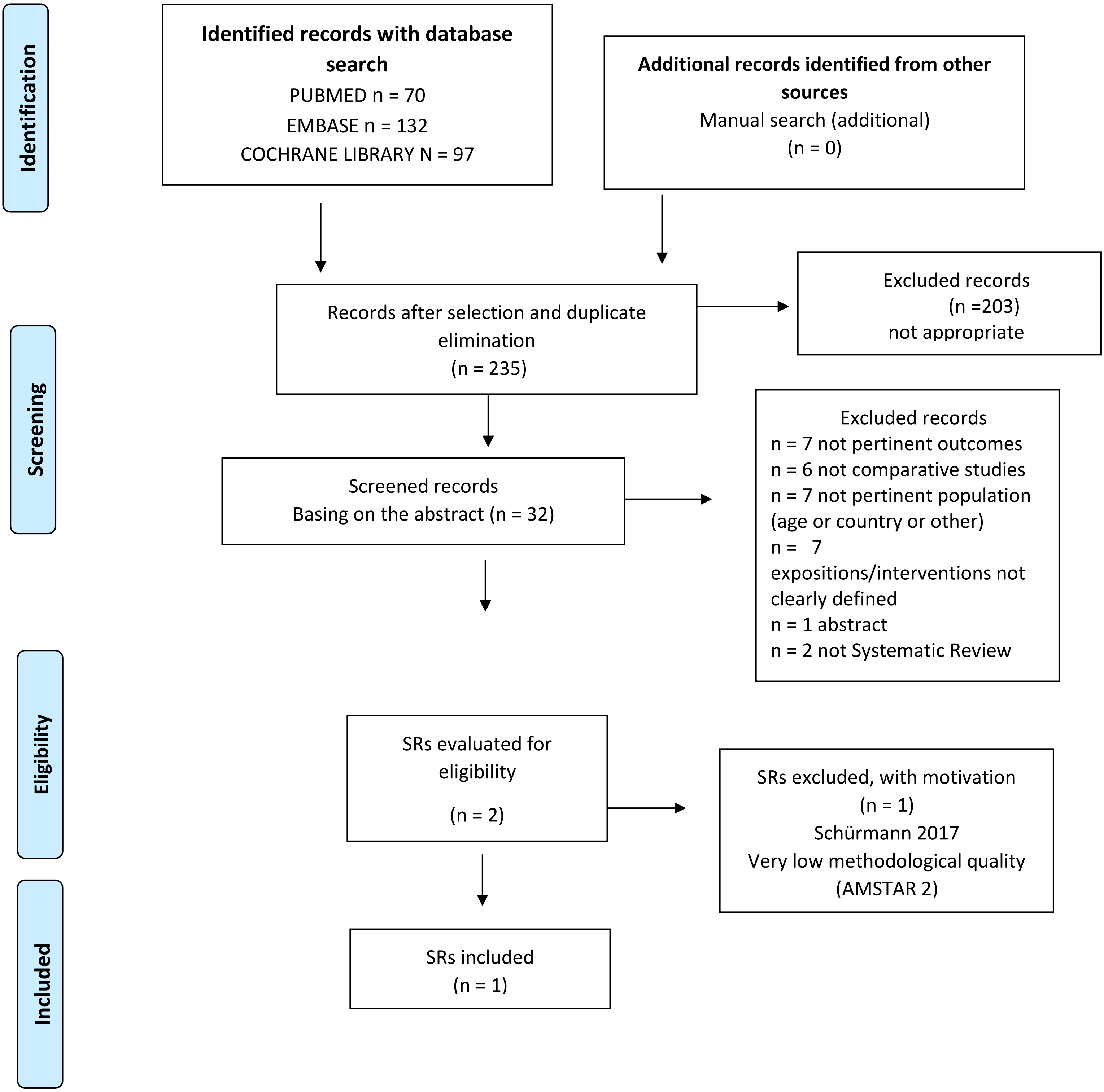

Figure S1c: Studies search flow diagram.

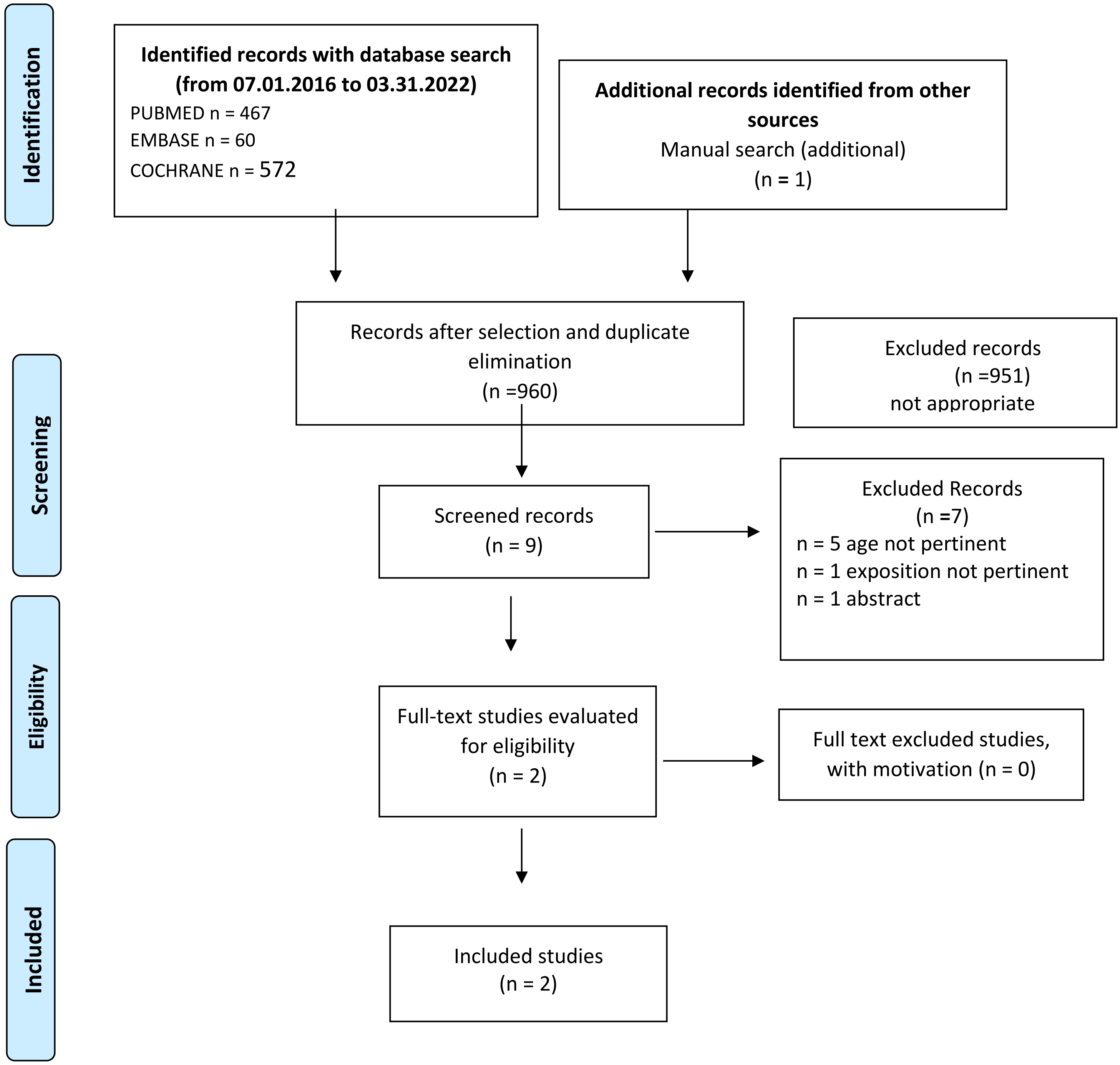

Key question

2. Does complementary feeding completely or partially free of animal-source foods in healthy infants/toddlers result in a psychomotor development that is significantly different from that of infants on a balanced omnivorous diet?

PICOs

P. Will a healthy infant at the beginning and during the complementary feeding period (6-24 months)
I. who is offered a diet completely or partially devoid of food of animal origin
C. towards an infant who, in the same period, is offered a balanced omnivorous diet
O. have significantly different psychomotor development?

Systematic Reviews search

COCHRANE LIBRARY

in Title Abstract Keyword (Word variations have been searched)

A. "Infant Nutritional Physiological Phenomena"
B. "complementary feeding"
C. " weaning"
D. "child neurodevelopment"
E. "brain development"
F. "cerebral development"

Custom date range Topics: **01.01.2012 - 31.03.2022**

PubMed

#1
((("diet, mediterranean"[MeSH Terms] OR "diet, healthy"[MeSH Terms] OR "diet, vegetarian"[MeSH Terms] OR "diet, western"[MeSH Terms] OR "dietary pattern*"[All Fields] OR "veg"[All Fields])) AND (((((("Language Development Disorders"[Mesh]) OR "Language Development"[Mesh]) OR "Learning Disabilities"[Mesh]) OR "Psychomotor Disorders"[Mesh] OR "Psychological Phenomena"[Mesh]) OR "neurodevelopmental disorders"[MeSH Terms]) OR "neurodevelopment")
Filters: Meta-Analysis, Systematic Review, in the last 10 years, Child: birth-18 years

EMBASE

1
('diet, vegetarian'/exp OR 'diet, vegetarian' OR 'plant-based diet'/exp OR 'plant-based diet' OR 'vegetarian diet'/exp OR 'vegetarian diet' OR 'vegetarian eating patterns'/exp OR 'vegetarian eating

patterns' OR 'vegetarianism'/exp OR 'vegetarianism') AND ('brain cortex development'/exp OR 'brain cortex development' OR 'brain development'/exp OR 'brain development' OR 'cerebral development'/exp OR 'cerebral development' OR 'development, brain'/exp OR 'development, brain' OR 'child development'/exp OR 'child development' OR 'development, child'/exp OR 'development, child' OR 'infant development'/exp OR 'infant development') AND ('child'/exp OR 'child' OR 'infant'/exp OR 'infant' OR 'preschool child'/exp OR 'preschool child' OR 'adolescent'/exp OR 'adolescent' OR 'teenager'/exp OR 'teenager') AND ([cochrane review]/lim OR [systematic review]/lim OR [meta analysis]/lim) AND [2012-2022]/py

APA PsycInfo

1.
diet mediterranean " OR " diet healthy " OR " diet vegetarian " OR " diet western AND Any Field: "Language Development Disorders" OR Any Field: "Language Development" OR Any Field: "Learning Disabilities" OR Any Field: "Psychomotor Disorders" OR Any Field: "neurodevelopmental disorders" OR Any Field: "neurodevelopment" AND Age Group: Childhood (birth-12 yrs) AND Age Group: Adolescence (13-17 yrs) AND Methodology: Systematic Review AND Methodology: Meta Analysis AND Year: 2012 To 2022

Studies search

Cochrane

("Infant Nutritional Physiological Phenomena" OR "complementary feeding" OR " weaning" OR “Diet, Vegetarian”) AND ("child neurodevelopment" OR "brain development" OR "cerebral development") in Title Abstract Keyword - with Cochrane Library publication date Between Jan 1982 and Jun 2022, in Trials (Word variations have been searched)

PubMed

#1

(("diet, mediterranean"[MeSH Terms] OR "diet, healthy"[MeSH Terms] OR "diet, vegetarian"[MeSH Terms] OR "diet, western"[MeSH Terms] OR "dietary pattern*"[All Fields] OR "veg"[All Fields]) AND ("Language Development Disorders"[MeSH Terms] OR "Language Development"[MeSH Terms] OR "Learning Disabilities"[MeSH Terms] OR "Psychomotor Disorders"[MeSH Terms] OR "Psychological Phenomena"[MeSH Terms] OR "neurodevelopmental disorders"[MeSH Terms]) OR "neurodevelopment"))) AND ((casereports[Filter] OR clinicalstudy[Filter] OR clinicaltrial[Filter] OR clinicaltrialphaseiv[Filter] OR comparativestudy[Filter] OR controlledclinicaltrial[Filter] OR multicenterstudy[Filter] OR observationalstudy[Filter] OR pragmaticclinicaltrial[Filter] OR randomizedcontrolledtrial[Filter]) AND (1982/3/1:2022[pdat]) AND (allchild[Filter]))

EMBASE

#1

('diet, vegetarian'/exp OR 'diet, vegetarian' OR 'plant-based diet'/exp OR 'plant-based diet' OR 'vegetarian diet'/exp OR 'vegetarian diet' OR 'vegetarian eating patterns'/exp OR 'vegetarian eating patterns' OR 'vegetarianism'/exp OR 'vegetarianism') AND ('brain cortex development'/exp OR 'brain cortex development' OR 'brain development'/exp OR 'brain development' OR 'cerebral development'/exp OR 'cerebral development' OR 'development, brain'/exp OR 'development, brain' OR 'child development'/exp OR 'child development' OR 'development, child'/exp OR 'development, child' OR 'infant development'/exp OR 'infant development') AND ('child'/exp OR 'child' OR 'infant'/exp OR 'infant' OR 'preschool child'/exp OR 'preschool child' OR 'adolescent'/exp OR 'adolescent' OR 'teenager'/exp OR 'teenager') AND ('case report'/de OR 'case control study'/de OR 'clinical trial'/de OR 'cohort analysis'/de OR 'comparative study'/de OR 'controlled clinical trial'/de OR 'controlled study'/de OR 'cross sectional study'/de OR 'intervention study'/de OR 'longitudinal study'/de OR 'multicenter study'/de OR 'observational study'/de OR 'prospective study'/de OR 'randomized controlled trial'/de OR 'randomized controlled trial topic'/de OR 'retrospective study'/de) AND [1982-2022]/py

APA PsycInfo

1

Any Field: diet mediterranean " OR " diet healthy " OR " diet vegetarian " OR " diet western *AND* Any Field: Any Field: "Language Development Disorders" *OR* Any Field: Any Field: "Language Development" *OR* Any Field: Any Field: "Learning Disabilities" *OR* Any Field: Any Field: "Psychomotor Disorders" *OR* Any Field: Any Field: "neurodevelopmental disorders" *OR* Any Field: Any Field: "neurodevelopment" *AND* Year: 1982 *To* 2022

Figure S1d: SRs search flow diagram

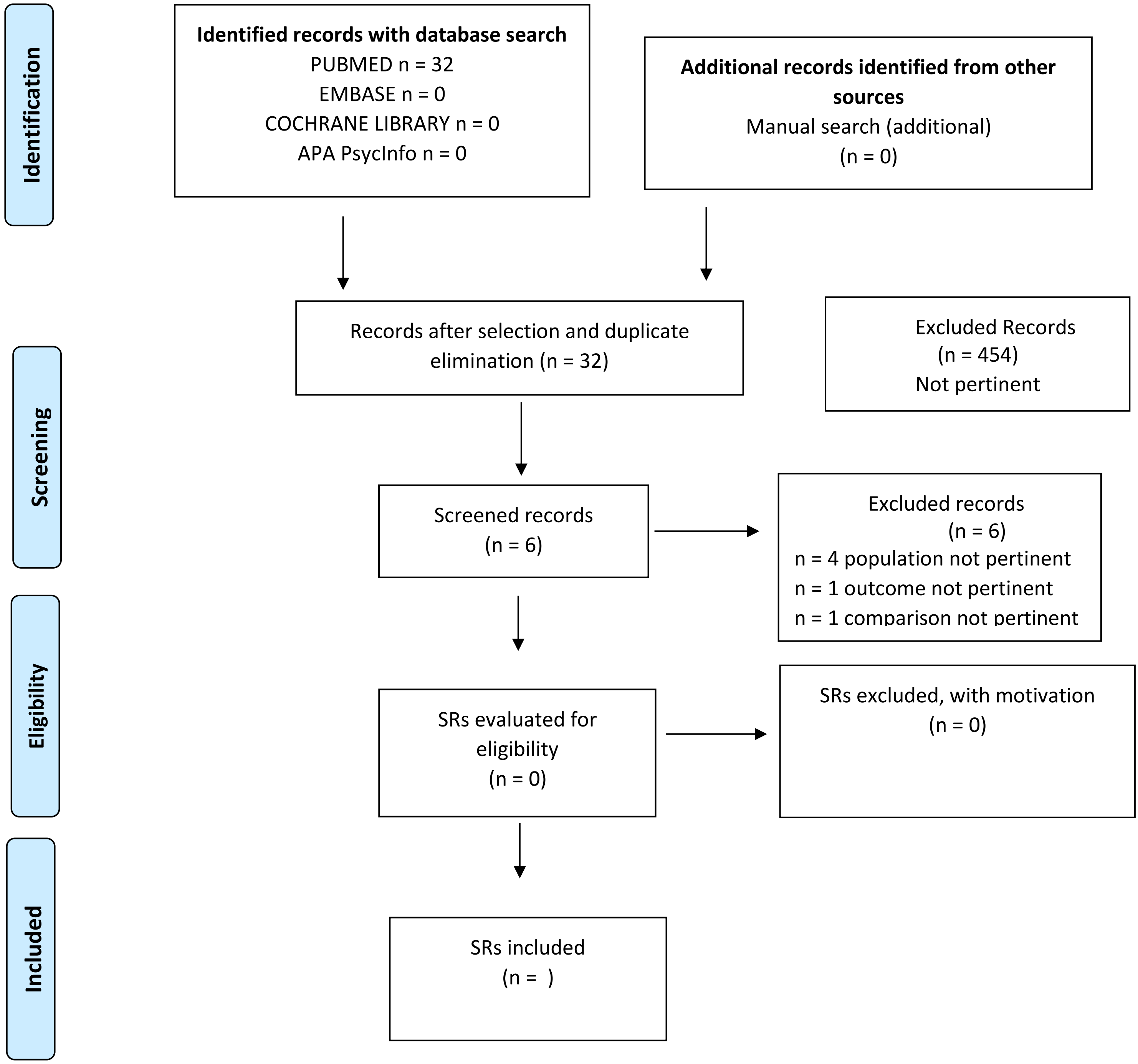

Figure S1e: Studies search flow diagram.

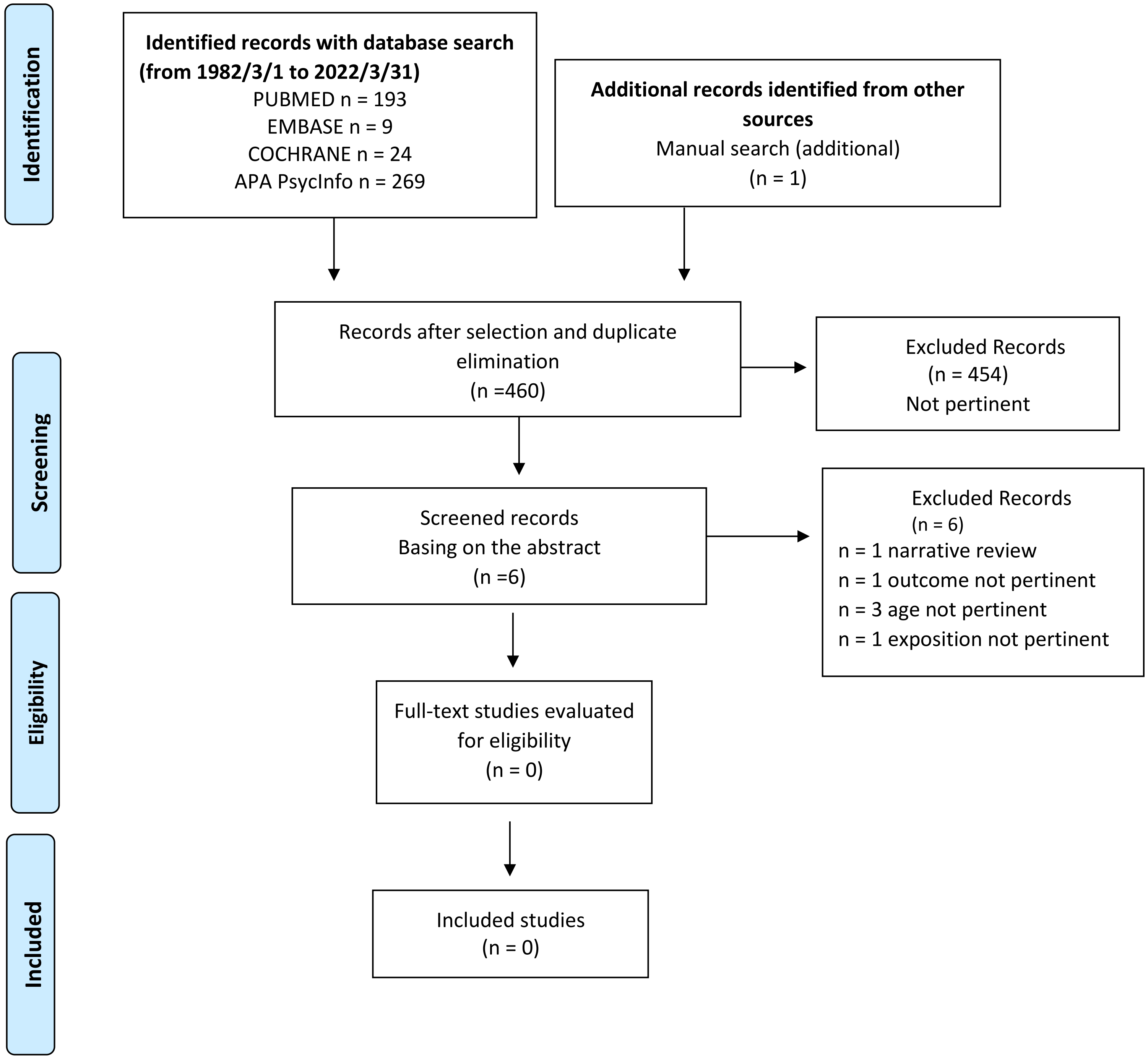

Key questions

3. Do healthy infants on a complementary feeding that is completely or partially free of any animal-based foods
    3.1. have a different risk of developing NCDs (Obesity/Overweight/Hypertension, Diabetes Mellitus)?

    compared to infants on a balanced omnivorous diet?

PICOs

3.1.

P. Will a healthy infant at the beginning and during the complementary feeding period (6-24 months)
I. who is offered a diet completely or partially devoid of food of animal origin
C. towards an infant who, in the same period, is offered a balanced omnivorous diet
O. have a different risk of developing NCDs (Obesity / Overweight, Hypertension, Diabetes mellitus)?

Systematic Reviews search

COCHRANE LIBRARY

“Noncommunicable Diseases”

“Diabetes”

“Hypertension”

"Infant Nutritional Physiological Phenomena"

" weaning"

“Diet, Vegetarian”

"complementary feeding”

in Title Abstract Keyword (Word variations have been searched)

Custom date range Topics: **01.01.2012 - 03.31.2022**

PubMed

#1

(("Hypertension"[MeSH Terms] OR "arterial hypertension"[All Fields] OR "elevated blood pressure"[All Fields] OR "Diabetes Mellitus"[MeSH Terms] OR "diabetes mellitus/prevention and control"[MeSH Terms] OR "Glucose Intolerance"[MeSH Terms] OR "Hyperglycemia"[MeSH Terms] OR "Noncommunicable Diseases"[MeSH Terms]) AND ("diet, mediterranean"[MeSH Terms] OR "diet, healthy"[MeSH Terms] OR "diet, vegetarian"[MeSH Terms] OR "diet, western"[MeSH Terms] OR "dietary pattern*"[All Fields] OR "veg"[All Fields])) AND ((y_10[Filter]) AND (meta-analysis[Filter] OR systematicreview[Filter]) AND (allchild[Filter]))

EMBASE

#1

('diet, vegetarian'/exp OR 'diet, vegetarian' OR 'plant-based diet'/exp OR 'plant-based diet' OR 'vegetarian diet'/exp OR 'vegetarian diet' OR 'vegetarian eating patterns'/exp OR 'vegetarian eating patterns' OR 'vegetarianism'/exp OR 'vegetarianism') AND ('non communicable disease'/exp OR 'diabetes mellitus'/exp OR 'hyperglycemia'/exp OR ‘hypertension'/exp OR 'blood pressure'/exp OR 'elevated blood pressure'/exp) AND ('child'/exp OR 'child' OR 'infant'/exp OR 'infant' OR 'preschool child'/exp OR 'preschool child' OR 'adolescent'/exp OR 'adolescent' OR 'teenager'/exp OR 'teenager') AND ([cochrane review]/lim OR [systematic review]/lim OR [meta analysis]/lim) AND [2012-2022]/py

Studies search

Cochrane

(“Noncommunicable Diseases” OR “Diabetes” OR “Hypertension”) AND ("Infant Nutritional Physiological Phenomena" OR "complementary feeding" OR " weaning" OR “Diet, Vegetarian”) in Title Abstract Keyword - with Cochrane Library publication date Between Jan 1982 and Jun 2022 (Word variations have been searched)

PubMed

#1

(("diet, mediterranean"[MeSH Terms] OR "diet, healthy"[MeSH Terms] OR "diet, vegetarian"[MeSH Terms] OR "diet, western"[MeSH Terms] OR "dietary pattern*"[All Fields] OR "veg"[All Fields]) AND ("Hypertension"[MeSH Terms] OR "arterial hypertension"[All Fields] OR "elevated blood pressure"[All Fields] OR "Diabetes Mellitus"[MeSH Terms] OR "diabetes mellitus/prevention and control"[MeSH Terms] OR "Glucose Intolerance"[MeSH Terms] OR "Hyperglycemia"[MeSH Terms] OR "Noncommunicable Diseases"[MeSH Terms])) AND ((casereports[Filter] OR clinicalstudy[Filter] OR clinicaltrial[Filter] OR clinicaltrialphaseiv[Filter] OR comparativestudy[Filter] OR controlledclinicaltrial[Filter] OR multicenterstudy[Filter] OR observationalstudy[Filter] OR pragmaticclinicaltrial[Filter] OR randomizedcontrolledtrial[Filter]) AND (1982/3/1:2022/3/22[pdat]) AND (allchild[Filter]))

EMBASE

#1

('diet, vegetarian'/exp OR 'diet, vegetarian' OR 'plant-based diet'/exp OR 'plant-based diet' OR 'vegetarian diet'/exp OR 'vegetarian diet' OR 'vegetarian eating patterns'/exp OR 'vegetarian eating patterns' OR 'vegetarianism'/exp OR 'vegetarianism') AND ('non communicable disease'/exp OR 'diabetes mellitus'/exp OR 'hyperglycemia'/exp OR ‘hypertension'/exp OR 'blood pressure'/exp OR 'elevated blood pressure'/exp) AND ('child'/exp OR 'child' OR 'infant'/exp OR 'infant' OR 'preschool child'/exp OR 'preschool child' OR 'adolescent'/exp OR 'adolescent' OR 'teenager'/exp OR 'teenager') # AND ('case report'/de OR 'case control study'/de OR 'clinical trial'/de OR 'cohort analysis'/de OR 'comparative study'/de OR 'controlled clinical trial'/de OR 'controlled study'/de OR 'cross sectional study'/de OR 'intervention study'/de OR 'longitudinal study'/de OR 'multicenter study'/de OR 'observational study'/de OR 'prospective study'/de OR 'randomized controlled trial'/de OR 'randomized controlled trial topic'/de OR 'retrospective study'/de) AND [1982-2022]/py

Figure S1f: SRs search flow diagram

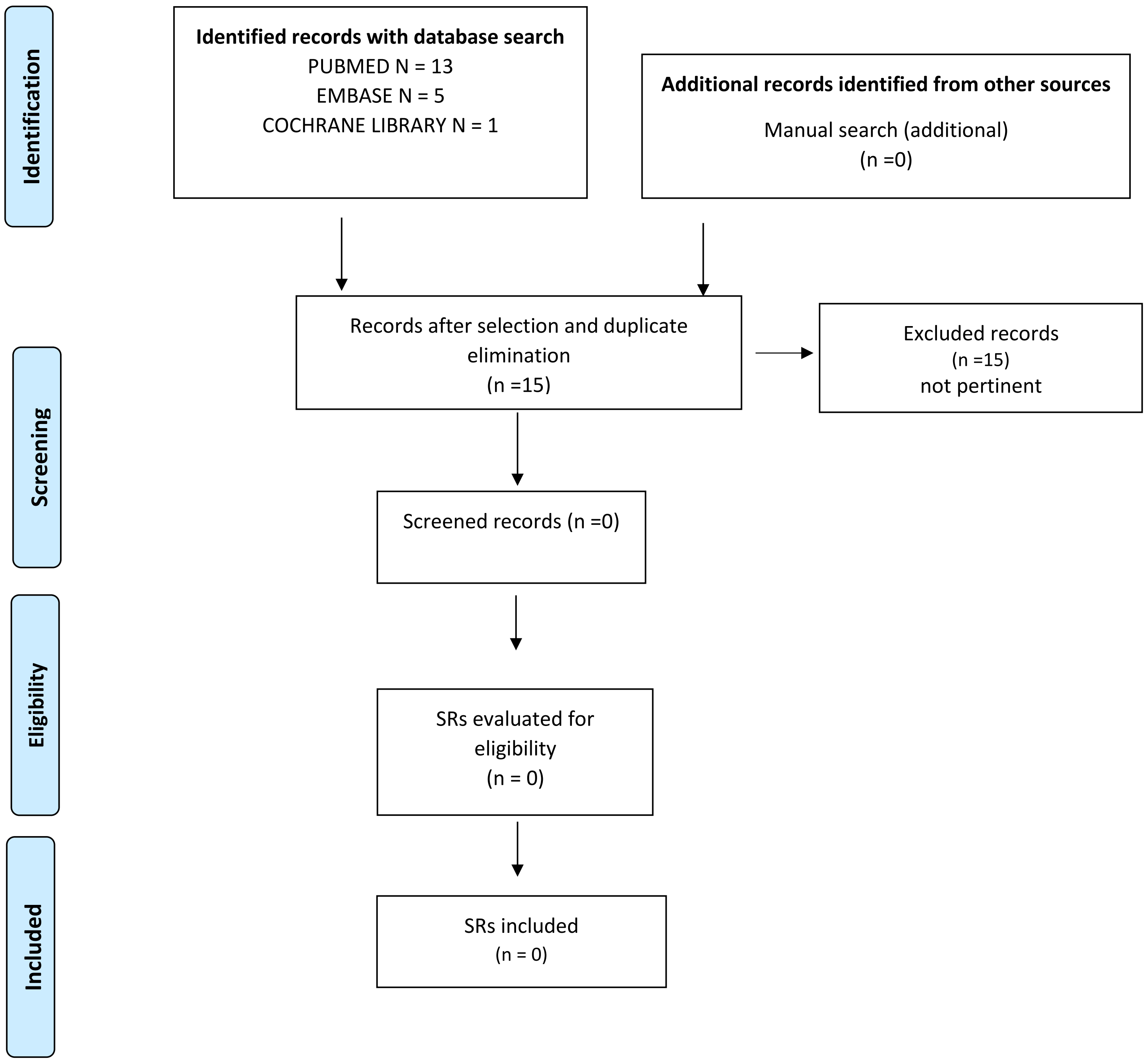

Figure S1g: Studies search flow diagram.

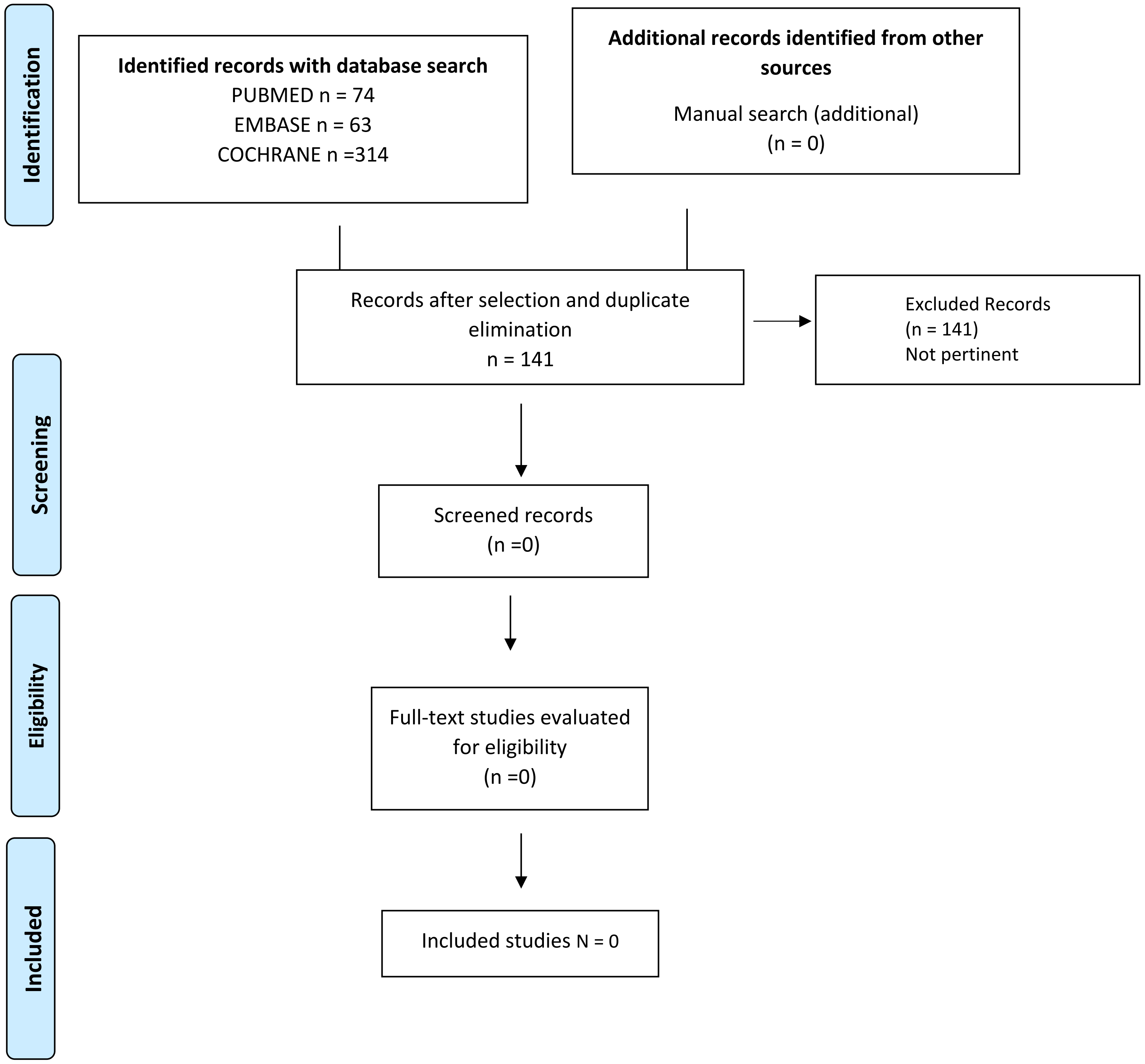

Key questions

3. Do healthy infants on a complementary feeding that is completely or partially free of any animal-based foods

    3.2. have a different risk of developing vitamin or other micronutrient deficiencies?

    compared to infants on a balanced omnivorous diet?

PICOs

3.2.

P. Will a healthy infant at the beginning and during the complementary feeding period (6-24 months)
I. who is offered a diet completely or partially devoid of food of animal origin
C. towards an infant who, in the same period, is offered a balanced omnivorous diet
O. have a different risk of developing vitamin or micronutrient deficiencies?

Systematic Reviews search

COCHRANE LIBRARY

"Infant Nutritional Physiological Phenomena"
" weaning"
“Diet, Vegetarian”
"complementary feeding”
“Micronutrients”
“malnutrition”
"Iron Deficiencies"
“Anemia”
in Title Abstract Keyword (Word variations have been searched)

<u>Custom date range Topics</u>: 01.01.2012 - 03.31.2022

PubMed

#1
((("Iron"[Mesh] "Iron Deficiencies"[MeSH Terms] OR "Malnutrition"[MeSH Terms]* OR "Micronutrients"[MeSH Terms] OR "micronutrients deficiency"[All Fields]) AND ("diet, mediterranean"[MeSH Terms] OR "diet, healthy"[MeSH Terms] OR "diet, vegetarian"[MeSH Terms] OR "diet, western"[MeSH Terms] OR "dietary pattern*"[All Fields] OR "veg"[All Fields])) AND ((y_10[Filter]) AND (meta-analysis[Filter] OR systematicreview[Filter]) AND (allchild[Filter]))

EMBASE

#1
'vegetarian diet'/exp OR 'vegetarian diet' OR (('vegetarian'/exp OR vegetarian) AND ('diet'/exp OR diet))AND 'nutritional deficiency'/exp' OR vitamin deficiency'/exp OR 'vitamin deficiency' OR 'anemia'/exp OR 'iron deficiency anemia'/exp AND ([cochrane review]/lim OR [systematic review]/lim OR [meta analysis]/lim) AND ([infant]/lim OR [child]/lim OR [preschool]/lim OR [school]/lim OR [adolescent]/lim) AND [2012-2022]/py

Studies search

Cochrane

("Infant Nutritional Physiological Phenomena" OR "complementary feeding" OR " weaning" OR “Diet, Vegetarian”) AND (“Micronutrients” OR “malnutrition” OR "Iron Deficiencies" OR "anemia")
in Title Abstract Keyword - with Cochrane Library publication date Between Jan 1982 and Jun 2022, in Trials (Word variations have been searched)

PubMed

#1
((("Iron"[Mesh] OR "Iron Deficiencies"[MeSH Terms] OR "Malnutrition"[MeSH Terms]* OR "Micronutrients"[MeSH Terms] OR "micronutrients deficiency"[All Fields]) AND ("diet, mediterranean"[MeSH Terms] OR "diet, healthy"[MeSH Terms] OR "diet, vegetarian"[MeSH Terms] OR "diet, western"[MeSH Terms] OR "dietary pattern*"[All Fields] OR "veg"[All Fields])) AND ((casereports[Filter] OR clinicalstudy[Filter] OR clinicaltrial[Filter] OR clinicaltrialphaseiv[Filter] OR comparativestudy[Filter] OR controlledclinicaltrial[Filter] OR multicenterstudy[Filter] OR observationalstudy[Filter] OR pragmaticclinicaltrial[Filter] OR randomizedcontrolledtrial[Filter]) AND (1982/3/1:2022/3/20[pdat]) AND (allchild[Filter]))

EMBASE

#1
('case report'/de OR 'case control study'/de OR 'clinical trial'/de OR 'cohort analysis'/de OR 'comparative study'/de OR 'controlled clinical trial'/de OR 'controlled study'/de OR 'cross sectional study'/de OR 'intervention study'/de OR 'longitudinal study'/de OR 'multicenter study'/de OR 'observational study'/de OR 'prospective study'/de OR 'randomized controlled trial'/de OR 'randomized controlled trial topic'/de OR 'retrospective study'/de) AND [**1982-2022]**/py AND ('vegetarian diet'/exp OR 'diet'/exp) AND ('nutritional deficiency'/exp OR 'vitamin deficiency'/exp

OR 'iron deficiency anemia'/exp) AND ([adolescent]/lim OR [child]/lim OR [infant]/lim OR [preschool]/lim OR [school]/lim)

Figure S1h: SRs search flow diagram

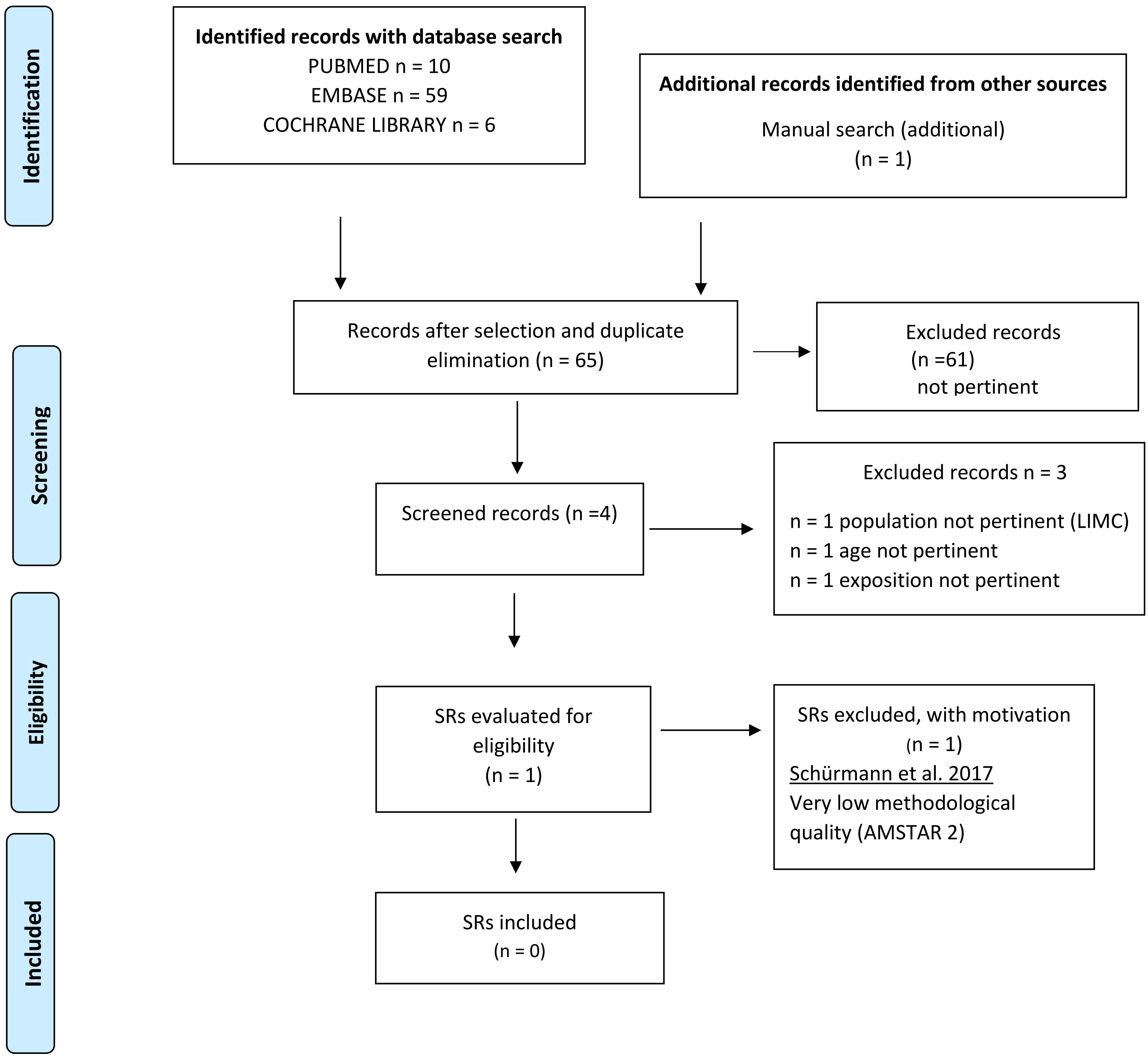

Figure S1i: Studies search flow diagram.

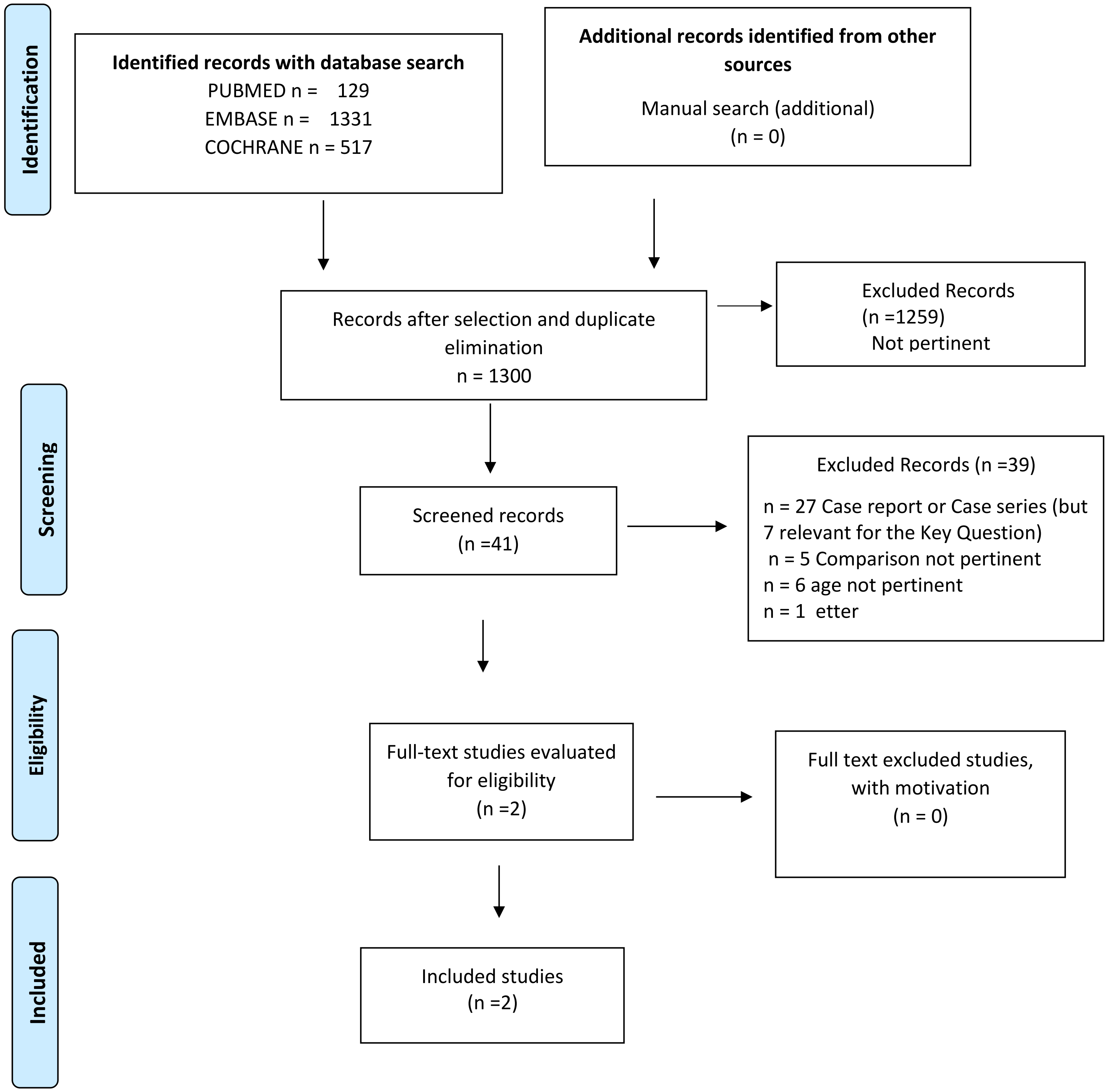

Key questions

3. Do healthy infants on a complementary feeding that is completely or partially free of any animal-based foods

    3.3. have a different risk of developing infections and related outcomes

compared to infants on a balanced omnivorous diet?

PICOs

3.3.

P. Will a healthy infant at the beginning and during the complementary feeding period (6-24 months)
I. who is offered a diet completely or partially devoid of food of animal origin
C. towards an infant who, in the same period, is offered a balanced omnivorous diet
O. have will it have a different risk of developing infections and related outcomes?

Systematic Reviews search

COCHRANE LIBRARY

"Infant Nutritional Physiological Phenomena"
"complementary feeding"
"weaning"
“Diet, Vegetarian”
"Infections"
in Title Abstract Keyword - with Cochrane Library publication date Between Jan 2012 and Jun 2022, in Trials (Word variations have been searched)

PubMed

#1

("Infections"[MeSH Terms] AND ("diet, mediterranean"[MeSH Terms] OR "diet, healthy"[MeSH Terms] OR "diet, vegetarian"[MeSH Terms] OR "diet, western"[MeSH Terms] OR "dietary pattern*"[All Fields] OR "veg"[All Fields])) AND ((y_10[Filter]) AND (meta-analysis[Filter] OR systematicreview[Filter]))

EMBASE

#1

('vegetarian diet'/exp OR 'diet'/exp) AND 'infection'/exp AND ([cochrane review]/lim OR [systematic review]/lim OR [meta analysis]/lim) AND [2012-2022]/py AND ([child]/lim OR [infant]/lim OR [preschool]/lim)

Studies search

Cochrane

("Infant Nutritional Physiological Phenomena" OR "complementary feeding" OR " weaning" OR “Diet, Vegetarian”) AND "infections"
in Title Abstract Keyword - in Trials with Cochrane Library publication date Between Jan 1982 and Jun 2022 (Word variations have been searched)

PubMed

#1

("Infections"[MeSH Terms] AND ("diet, mediterranean"[MeSH Terms] OR "diet, healthy"[MeSH Terms] OR "diet, vegetarian"[MeSH Terms] OR "diet, western"[MeSH Terms] OR "dietary pattern*"[All Fields] OR "veg"[All Fields])) AND ((clinicaltrial[Filter] OR comparativestudy[Filter] OR controlledclinicaltrial[Filter] OR multicenterstudy[Filter] OR observationalstudy[Filter] OR pragmaticclinicaltrial[Filter] OR randomizedcontrolledtrial[Filter]) AND (1982:2022[pdat]) AND (allinfant[Filter]))

EMBASE

#1

('vegetarian diet'/exp OR 'diet'/exp) AND 'infection'/exp AND ([infant]/lim OR [preschool]/lim) AND [1982-2022]/py

Figure S1j: SRs search flow diagram

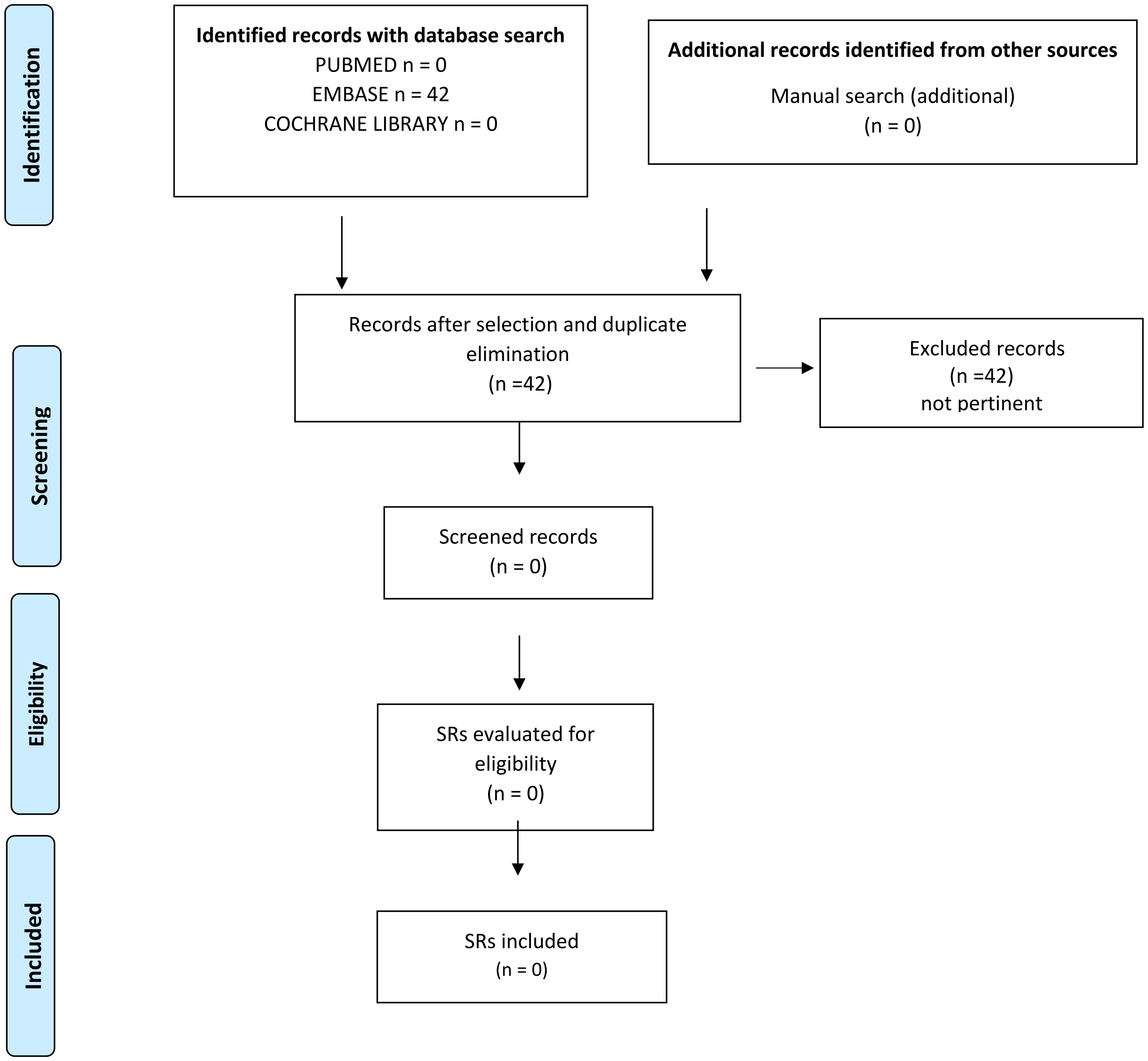

Figure S1k: Studies search flow diagram.

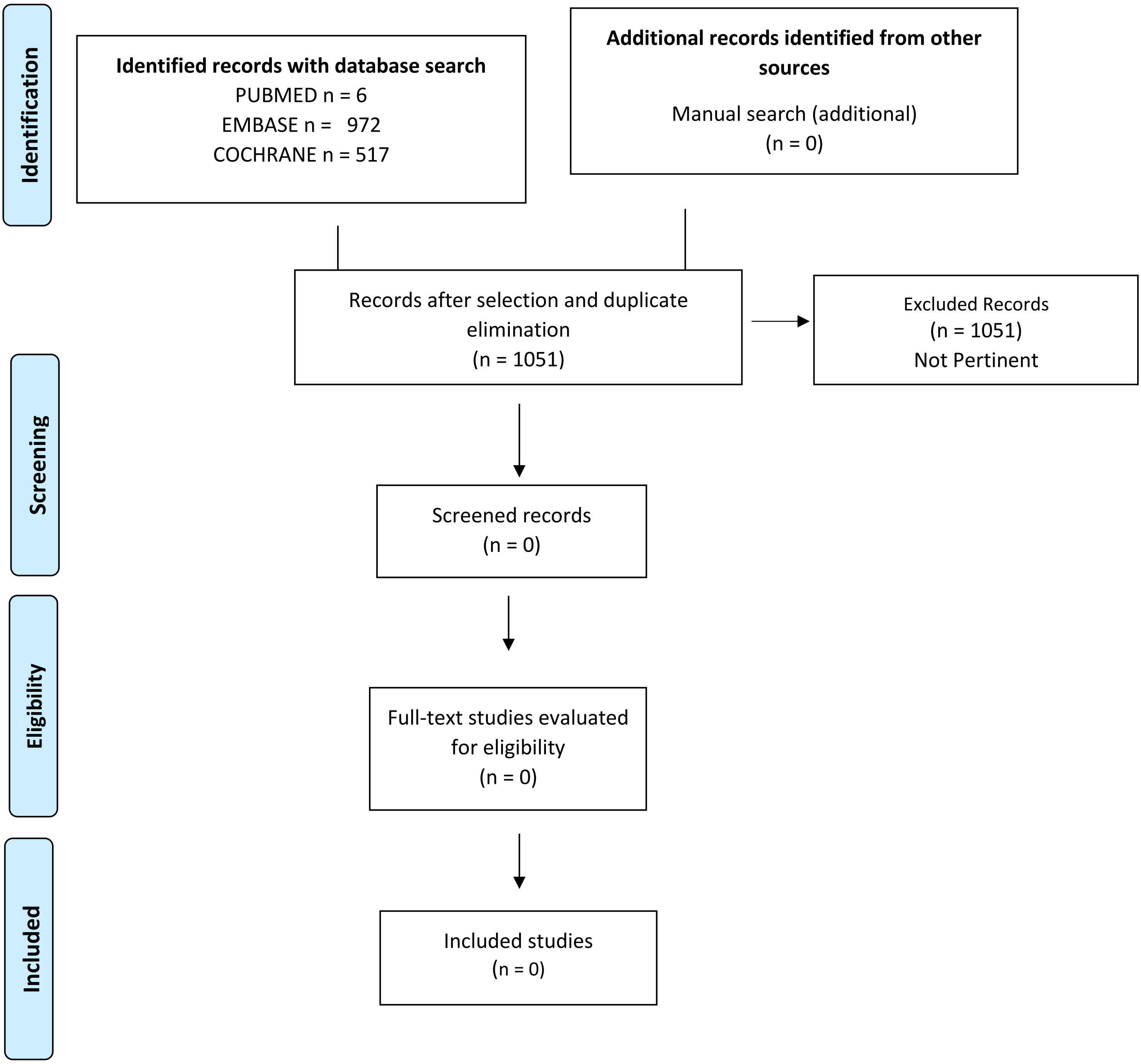

Supplement: Supplementary file 1 [file nutrients-14-03591-s001.zip › nutrients_1869505_Supplementary file S1.pdf]
