# Supplementary material for: Do Vegetarian Diets Provide Adequate Nutrient Intake during Complementary Feeding? A Systematic Review
Source: Nutrients. 2022 Aug 31;14(17):3591. doi: 10.3390/nu14173591 (PMC9459879; doi:10.3390/nu14173591)
Supplement: Supplementary file 1 [file nutrients-14-03591-s001.zip › nutrients_1869505_Supplementary file S2.pdf]

File S2. METHODOLOGICAL ASSESSMENT

Table S2a: Clinical Guidelines and Documents included.

Included Document	Multidisciplinarity of the panel	Systematic search for evidence	Grading of recommendations
Position Paper SIPPS - FIMP - SIMA – SIMP 2017 [23]	Yes	Yes	Yes

Table S2b: Clinical Guidelines and Documents excluded.

Excluded GLs	Multidisciplinarity of the panel	Systematic search for evidence	Grading of recommendations	Reason for exclusion
Fewtrell et al. 2017 [67]	Unclear	No	No	Very low quality
Agnoli et al. 2017 [68]	Yes but there is a lack of important experts/stakeholders as specialists in paediatrics and gynaecology	Declared and briefly described but not reported. The results do not report the methodological quality of the evidence	No	Low quality
U.S. Department of Agriculture and U.S. Department of Health and Human Services. Dietary Guidelines for Americans, 2020-2025. [69, 70]	Yes	Yes	No<br>Grading of evidence	Evaluate the intakes of the lacto-egg-vegetarian diet compared to the needs of children aged 12-24 months. It does not evaluate vegetarian diets in children aged 6-12 months. It does not report pertinent recommendations to the KQs of this SR
Redecilla Ferreiro et al. 2020 [71]	Unclear	No	No	Very low quality
Lemale et al. 2019 [72]	No	No	No	Very low quality

Table S2c: Appraisal of the Systematic Review

**AMSTAR 2**	English et al. 2019 [41]
**1. Did the research questions and inclusion criteria for the review include the components of PICO? (Yes/No)**	No
**2. Did the report of the review contain an explicit statement that the review methods were established before the conduct of the review and did the report justify any significant deviations from the protocol? (Yes/Partial Yes/No)**	Partial yes (*there was an analytic framework*)
**3. Did the review authors explain their selection of the study designs for inclusion in the review? (Yes/No)**	No
**4. Did the review authors use a comprehensive literature search strategy? (Yes/Partial Yes/No)**	No<br>Publication restrictions (only english language) not justified<br>Search not conducted within 24 months of completion of the review
**5. Did the review authors perform study selection in duplicate? (Yes/No)**	Yes
**6. Did the review authors perform data extraction in duplicate? (Yes/No)**	Yes
**7. Did the review authors provide a list of excluded studies and justify the exclusions? (Yes/Partial Yes/No)**	Yes
**8. Did the review authors describe the included studies in adequate detail? (Yes/Partial Yes/No)**	Yes
**9. Did the review authors use a satisfactory technique for assessing the risk of bias (RoB) in individual studies that were included in the review? (Yes/Partial Yes/No/Includes only NRSI-RCT)**	Yes<br>Yes
**10. Did the review authors report on the sources of funding for the studies included in the review? (Yes/No)**	No

**11. If meta-analysis was performed did the review authors use appropriate methods for statistical combination of results?**<br>**(Yes / No / No meta-analysis conducted)**	No meta-analysis conducted
**12. If meta-analysis was performed, did the review authors assess the potential impact of RoB in individual studies on the results of the meta-analysis or other evidence synthesis?**<br>**(Yes / No / No meta-analysis conducted)**	No meta-analysis conducted
**13. Did the review authors account for RoB in individual studies when interpreting/ discussing the results of the review?**<br>**(Yes/No)**	Yes
**14. Did the review authors provide a satisfactory explanation for, and discussion of, any heterogeneity observed in the results of the review?**<br>**(Yes/No)**	Yes
**15. If they performed quantitative synthesis did the review authors carry out an adequate investigation of publication bias (small study bias) and discuss its likely impact on the results of the review?**<br>**(Yes / No / No meta-analysis conducted)**	No meta-analysis conducted
**16. Did the review authors report any potential sources of conflict of interest, including any funding they received for conducting the review?**<br>**(Yes/No)**	Yes
**OVERALL EVALUATION**	LOW QUALITY

Table S2d: SRs excluded with motivation.

**EXCLUDED**	**Reason for exclusion**
Schürmann et al. 2017 [9]	Very low quality (AMSTAR 2)

Table S2e: Appraisal of the Studies

Newcastle Quality Assessment Scale
COHORT STUDIES

	Selection				Comparability	Outcome				
**Study**	**Representativeness of the exposed cohort**	**Selection of non-exposed cohort**	**Ascertainment of the exposure**	**Demonstration that the outcome of interest is not present at the start of the study**	**Comparability of cohorts based on design or analysis**	**Outcome evaluation**		**Was the follow-up long enough for the outcome to occur?**	**Adequacy of cohort follow-up**	**Total**
Dagnelie et al. 1994 [42]	b*	a*	d	b	a*	b*		b	b*	5 *
Taylor et al. 2004 [53]	b*	a*	c	b	a,b**	b*		a*	c	6 *

Newcastle Quality Assessment Scale
CROSS-SECTIONAL STUDIES

	Selection				Comparability	Outcome		
**Study**	**Representativeness of the sample**	**Sample size**	**Non-Response rate**	**Ascertainment of exposure (max 2)**	**Comparability between groups, confounders are controlled (max2)**	**Outcome evaluation (max 2)**	**Statistical test**	**Total**
Weder et al. 2019 [43]	b*	b	b	b*	a,b**	c*	a*	6 *

Table S2f. Studies excluded with motivation.

**EXCLUDED**	**Reason for exclusion**
Sievers et a. 1991 [73]	Untraceable
