# Supplementary material for: Do Vegetarian Diets Provide Adequate Nutrient Intake during Complementary Feeding? A Systematic Review
Source: Nutrients. 2022 Aug 31;14(17):3591. doi: 10.3390/nu14173591 (PMC9459879; doi:10.3390/nu14173591)
Supplement: Supplementary file 1 [file nutrients-14-03591-s001.zip › nutrients_1869505_Supplementary file S3.pdf]

File S3. RECOMMENDATIONS OF THE GLs, RESULTS OF THE SRs AND STUDIES

Growth

1. Does complementary feeding completely or partially free of animal-source foods in healthy infants result in significantly different auxological development and/or growth compared with that of infants on a balanced omnivorous diet?	**P.** Will a healthy infant at the beginning and during the complementary feeding period (6-24 months)<br>**I.** who is offered a diet completely or partially devoid of food of animal origin<br>**C.** towards an infant who, in the same period, is offered a balanced omnivorous diet<br>**O.** have significantly different growth and development?

Table S3a. Included Guidelines and other Documents: Recommendations and Grading

Guidelines – other Documents	Quality of evidence	Recommendations	Grading
Position Paper SIPPS - FIMP - SIMA – SIMP 2017 [23]	very low: 4 observational studies were included, but none of them answered the question	Given the very low level of evidence, it is not possible to state with certainty that vegetarian diets in childhood and adolescence ensure adequate growth and nutritional status.<br>Given the very low level of evidence, it is not possible to establish at what age a vegetarian diet can be started without side effects on growth.<br>Quite the contrary, evidence does exist on the need to supplement diets that exclude certain food groups. The more restricted a diet is, the greater nutritional deficiencies are.<br>Specific nutritional counseling is recommended for appropriate supplementation, with particular reference to the amino acid profile of proteins and intakes of iron, zinc, vitamin B12 and DHA (strong positive recommendation).<br>Periodic nutritional status assessments in relation to supplementation in both children and adolescents are recommended	strong positive recommendation

Table S3b: Included SRs. Characteristics, Results, and Conclusions

Systematic Review	Population and purpose of the SR	Results	Conclusions
English et al. 2019 (SR of RCTs and observational studies) [41]	Population: Generally healthy children fed human milk, infant formula or both, with growth, size and body composition examined through adulthood<br><br>Purpose: To assess the relation between types and amount of complementary food and beverage consumed and growth, size and body composition.	Twenty-four of the 49 articles reported one or more significant associations, either positive, negative, or mixed, between types or amounts of CFB and growth, size, body composition, and/or risk of malnutrition, overweight or obesity.<br><br>Twenty-five of the 49 articles found no relationship between types and amounts of CFB and growth, size, body composition, and/or risk of overweight or obesity	- Moderate evidence indicates that higher versus lower meat intake or meat versus iron-fortified cereal intake over a short duration (~3mo) during the complementary feeding period does not favorably or unfavorably influence growth, size, and/or body composition.<br><br>- Moderate evidence suggests that consumption of complementary foods with different fats and/or fatty acid composition does not favorably or unfavorably influence growth, size, or body composition.<br><br>- Limited evidence suggests that type or amount of cereal given does not favorably or unfavorably influence growth, size, body composition, and/or prevalence/incidence of overweight or obesity.<br><br>- Limited evidence suggests that sugar-sweetened beverage consumption during the complementary feeding period is associated with increased risk of obesity in childhood, but is not associated with other measures of growth, size, and body composition.<br><br>- Limited evidence showed a positive association between juice intake and infant weight-for-length and child BMI z-scores.<br><br>- There is not enough evidence to determine a relationship between consumption of complementary foods with different fats and/or fatty acid composition and/or prevalence/incidence of overweight or obesity or to determine a relationship between meat intake and prevalence/incidence of overweight or obesity.<br><br>- For other complementary foods (vegetables, fruit, dairy products and/or cow's milk, cereal-based products, milk-cereal drink, and/or categories such as "ready-made foods") or

			distinct dietary patterns during the complementary feeding period no conclusion could be made about the relationship with growth, size, body composition, and/or prevalence/incidence of overweight or obesity.

Table S3c: Included studies. Characteristics and Results

Study (First Author, Year, Country/Setting)	Study design	Population (sample size, baseline characteristics)	Intervention/exposure	Primary Outcome	Measures of treatment effect	Secondary Outcomes	Follow-up	Results	Funding
Dagnelie et al. 1994 [42]<br>The Netherlands	cross sectional study followed by mixed-longitudinal study3.	N 243 (0-8 years) 2 years later same cohort N 194<br><br>study 2.<br>mixed longitudinal cohort study:<br><br>3 cohorts 4-10mo, 8.14 mo, 12-18 mo<br>white children with birth weight ≥2500 g and who did not suffer from a congenital disease. Children and their mothers should have been on a macrobiotic diet from birth of the child onward (n = 53). Omnivorous control subjects (n = 57)	Three cohorts of infants were monitored.<br>During this period, data on dietary intake, growth, blood chemistry, psycho-motor development, and physical deficiency symptoms were collected.<br><br>Intervention study: Intensive consultations with macrobiotic teachers in The Netherlands and abroad, were initiated. As a result, a joint report was handed out to all parents participating in the study<br><br>complementary feeding of macrobiotic infants started at 4. 8 mo$_{o}$. In the control group, complementary feeding started with fruits at mean age 2.7 mo	Cases of malnutrition in macrobiotic children incidental, or ubiquitous deficiencies	Longitudinal growth, expressed as change in SD scores (SDS) per year, of anthropometric indexes<br><br>-Anthropometric measurements including weight, height, sitting height, arm circumference, and tricipital and subscapular skinfold thicknesses	To provide recommendations for improving children's nutritional status that are acceptable within the macrobiotic philosophy	follow-up 2 y later supported this trend observed cross-sectionally<br><br>-	Longitudinal cohort study: Cross-sectional and mixed-longitudinal anthropometric studies in children from 0- 10 y of age revealed significantly depressed birth weight and marked growth retardation during the weaning period (6- 18 mo), followed by limited catch-up growth in weight and arm circumference, but there was no catch-up growth in height or sitting height.<br><br>Children from families that had increased the consumption of animal products had grown in height more rapidly	School of Psychology University of Nottingham (Within the last 5 years, ET and NP have received co-funding from Nutricia/Danone to support an ESRC CASE PhD studentship)

		study 3. Intervention study: Macrobiotic infants with the most clear-cut nutritional deficiencies (n = 27 from the original sample of 53 infants)							
Weder et al. 2019 [43] Germany	Cross-sectional study	430 children aged 1-3 years (127 vegetarian (VG), 139 vegan (VN), and 164 OM) in Germany with no diagnosed diseases that could affect the studied variables and that do not follow special diets other than vegan or vegetarian diets	A 3-day weighed dietary record assessed dietary intake, and an online questionnaire assessed lifestyle, body weight, and height. .	To compare the intake of energy, macronutrients, and fiber, as well as BW and BH, of VG, VN, and OM children aged 1–3 years in Germany.	Values are unadjusted arithmetic mean ± standard deviation (SD), whereas p-values and e ect sizes were derived from ANCOVA and adjusted for age and sex (basic model) or other confounders<br><br>Weight-for-height z-scores<br>Weight-for-Age z-score<br>Height-for-age z-scores		N.A.	There were no significant differences in energy intake or density and anthropometrics between the study groups.<br>Omnivorous children had the highest adjusted median intakes of protein, fat, and added sugars.<br>Vegan children had the highest adjusted intakes of carbohydrates and fibers.<br><br>A higher prevalence of children with inadequate growth was observed in the vegetarian and vegan diet groups: 21/266 VN and VG children were classified as stunted, i.e. with insufficient energy and long-term nutrient intake, or wasted, with severe malnutrition, compared to 1/164 OM children (OR [95% CI] = 13.97 [1.86, 104.88]; p=0.01) (Supplementary file S4, Analysis 1.2)	Not declared

Psychomotor development

2. Does complementary feeding completely or partially free of animal-source foods in healthy infants/toddlers result in a psychomotor development that is significantly different from that of infants on a balanced omnivorous diet?	**P.** Will a healthy infant at the beginning and during the complementary feeding period (6-24 months)<br>**I.** who is offered a diet completely or partially devoid of food of animal origin<br>**C.** towards an infant who, in the same period, is offered a balanced omnivorous diet<br>**O.** have significantly different psychomotor development?

Table S3.d. Included Guidelines and other Documents: Recommendations and Grading

Guidelines – other Documents	Quality of evidence	Recommendations	Grading
Position Paper SIPPS - FIMP - SIMA – SIMP 2017 [23]	No data available on the safety of CF without animal-based foods: scientific evidence only consists of case reports or case series<br><br>Low quality	It recommends close nutritional monitoring of the infant, even after the start of CF, with supplementation as needed to avoid severe clinical outcomes such as growth deficits, anaemia and especially neurological deficits	strong positive recommendation

Table S3e: Included studies. Characteristics and Results

Study (First Author, year, Country/Setting)	Study design	Population (sample size, baseline characteristics)	Intervention/exposure	Primary Outcome	Measure of effects	Secondary Outcomes	Follow-up	Results	Funding
Dagnelie et al. 1994 [42]<br>The Netherlands	cross sectional study followed by mixed-longitudinal study3.	N 243 (0-8 years)<br>2 years later same cohort N 194<br><br>study 2.<br>mixed longitudinal cohort study:<br><br>3 cohorts 4-10mo, 8.14 mo, 12-18 mo<br>white children with birth weight	Three cohorts of infants were monitored.<br>During this period, data on dietary intake, growth, blood chemistry, psycho- motor development, and physical deficiency symptoms were collected.<br><br>Intervention study: Intensive consultations	Cases of malnutrition in macrobiotic children incidental, or ubiquitous deficiencies	standard deviation scores	Psychomotor development appraised by means of a standardized checklist	2 years	The macrobiotic group was significantly slower in gross motor and, in speech and language development<br><br>Gross Motor Development<br>Sitting balance and head control SD = -0.48; p= 0.04<br>Walking SD= -0.60; p= 0.001<br>Overall score SD= -0.63; p < 0.001<br><br>Fine Motor Development SD= -0.13; p= 0.49	School of Psychology University of Nottingham (Within the last 5 years, ET and NP have received co-funding from Nutricia/Danone to support an ESRC CASE PhD studentship)

		≥2500 g and who did not suffer from a congenital disease. Children and their mothers should have been on a macrobiotic diet from birth of the child onward (n = 53). Omnivorous control subjects (n = 57)<br><br>study 3. Intervention study: Macrobiotic infants with the most clear-cut nutritional deficiencies (n = 27 from the original sample of 53 infants)	with macrobiotic teachers in The Netherlands and abroad, were initiated. As a result, a joint report was handed out to all parents participating in the study<br><br>complementary feeding of macrobiotic infants started at 4. 8 mo$_{o}$. In the control group, complementary feeding started with fruits at mean age 2.7 mo					Language Development SD= -0.42; p= 0.03	

Table S3f: Included Case reports. Characteristics and Results

CASE REPORT	Blasco-Alonso et al. [46]	Lemoine et al. [47]	Lund et al. [48]	Smolka et al. 2001 [49]	von Schenck et al. 1997 [50]	Lövblad et a.1997 [51]	Grattan-Smith et al. 1997 [52]
**AGE**	18 mo	13 mo	10 mo	Case 1. 13 mo<br>Case 2. 8 mo	14 mo	14,5 mo	Case 1. 10 mo<br>Case 2. 8 mo<br>Case 3. 18 mo
**DIET**	Breastfed from birth. His family has a vegetarian diet.	Prolonged breastfeeding from a vegetarian mother followed by a vegan diet for the infant after weaning.	Vegan diet without B12 supplementation	Dieta strattamente vegetariana	Vegan diet	Strictly vegetarian diet	Vegan diet
**SYMPTOMS**	Decreased level of consciousness and movement disorder (tremor and choreiform movements) of several hours' standing. History of delayed acquisition of motor milestones (not standing, monosyllabic language), and progressive loss of these over the last few weeks (head support and sitting)	Psychomotor regression , 4 bone fractures due to nutritional rickets. Weight at 2.7 SD (standard deviation), height at 1.4 SD, Waterlow score = 78%, and cranial circumference at 1.0 SD	Arrested development, passive behaviour and peripheral and truncal hypotonia. She could not sit, and she had reduced coordination, dyskinetic movements and generalised, tonic–clonic seizures	Case 1.<br>Psychomotor retardation, apathy, muscular hypotonia, abnormal movements, failure to thrive.<br><br>Caso 2. Psychomotor retardation, diskynesia, abnormal movements, failure to thrive.	anaemia and severe neurological abnormalities. unable to sit, became increasingly apathetic, and eventually stopped feeding, which provoked referral.<br>On admission he was comatose, had a high pitched cry and severe generalised muscular hypotonia.	Severe psychomotor retardation. Severely hypotonic child	Case 1.<br>Developmental regression, staring, and mouth smacking movements suggestive of seizures. Extremely lethargic, generally hypotonic.<br><br>Case 2.<br>Pale, irritable, lethargic, and constipated. rapid twitching movements around the mouth and tongue and of the orbicularis oculi.<br>She was irritable but alert and could fix and follow objects visually. When she reached out for toys, there was a marked tremor. She could not sit alone and could not support her weight. She was hypotonic<br><br>Case 3.<br>increasing hypotonia, persistent twitching movements of his right hand.. little spontaneous activity, but he would suck, cry, and roll over, There were continuous flexion-extension movements of his right hand involving the fingers and wrist.
**DIAGNOSTIC TESTS**	Cranial CT and MRI scans: generalized supratentorial atrophy involving both matters and the basal ganglia. Vitamin B12 d= 46 pg/ml (vn > 200), homocysteine = 125 mmol/L (vn < 10), urinary methylmalonic acid (154 mmol/L).	Normal calcium level = 2.21 mmol/L; normal values [N]: 2.1–2.6), hypophosphatemia (0.64 mmol/L; N: 1.3–1.8) with elevated alkaline phosphatase (ALP; (1066 IU/L; N < 35). Vitamin D deficiency (25- hydroxy-cholecalciferol: 22.6 nmol/L; N: 75–250) and secondary hyperparathyroidism	Hb= 1.8 mmol/L with megaloblastic changes, total p-homocysteine = 88 lmol/L (normal reference <15 lmol/L), p-methylmalonate = 17.7 lmol/L (<0.28 lmol/L), p-vitamin B12 <33 pmol/L (140–543 pmol/L). Cerebral imaging: mild central and cortical atrophy	Caso 1. Megaloblatic anemia, vitamin B12 levels = 35 pg / ml, methylmalnic aciduria = 451 mmol / mol. MRI: diffuse fronto-temporo-parietal atrophy and delayed myelination.<br><br>Case 2. Megaloblatic anemia, low levels of vitamin B12 = 51 pmol/L, methylmalnic	Severe dietary vitamin B-12 deficiency, macrocytic anaemia (haemoglobin 82 g/l, packed cell volume 0.245, mean corpuscular volume 116.7 fl). Serum ferritin was 4.0 μg/l, and red blood. Cranial magnetic resonance imaging (MRI) showed severe frontal and	Megaloblastic anemia, hemoglobin (Hb) = 6.0, erythrocytes=1.56 x 1012 , mean corpuscular Hb (MCHb)=39, hematocrit=0.17, MCV= 111, MCHC= 35. Vitamin B 12 = 92 pmol/l (normal value 180-750). MRI of the brain: severe brain atrophy with signs of retarded myelination, the frontal and temporal lobes being	Case 1.<br>Hemoglobin level= 10.5. GL, white blood cell count of 5.8 x 109/L, and platelets 430 x 109/L. His mean corpuscular volume (MCV) was 108 fl and mean corpuscular hemoglobin (MCH) level was 37 pg. The serum cobalamin level = 35 pmolL (normal range 150-700 pmol/L). Electroencephalogram (EEG) slowing of basic rhythms. (MR) imaging scan confirmed the atrophic changes and revealed delayed myelination, the appearance being more that of a 4-month-old infant.<br><br>Case 2.<br>Hemoglobin =7.8 gldl, cobalamin level =37 pmol/L (normal range 150-600 pmol/L).

		(21.2 pmol/L; N: 0.8–5.2). Bone X-rays: diffuse osteopenia with bone hypertransparency and fine cortical, consolidated left ulnar and bilateral peroneal diaphyseal fractures, enlargement, cupping, splaying, fraying and a coarse trabecular pattern in the metaphysis, with widening of the growth plate, and an enlargement of the anterior costochondral joints.		aciduria = 1578 μmol/L.<br>MRI: microcephaly.	frontoparietal cranial atrophy	most severely affected. Electroencephalogram: generalized slowing of the basal activity compatible with diffuse encephalopathy.	macrocytic film. Her serum cobalamin level was 37 pmol/L (normal range 150-600 pmol/L).<br><br>Case 3.<br>hemoglobin =12.1 g/dl,, serum cobalamin = 85 pmol/L (A CT scan of his head revealed marked cerebral atrophy<br>and bilateral subdural CSF collections with a larger<br>collection on the left, normal range 150-600 pmol/L).
**DIAGNOSIS THERAPY RESULTS**	Intramuscular vitamin B12 and then oral cyanocobalamin. Dietary diversification. Clinical normalization after 6 months , but with the need for rehabilitation treatment, and radiological normalization after 7 months	Vitamin D deficiency rickets, with no basis for hereditary rickets or other risk factors for developing rickets apart from diet. Therapy: vitamin D (100,000 IU/month for 3 months) and calcium (500 mg/day for 15 days). Diversified diet with animal proteins. General condition quickly improved, now developing well	Vitamin B12 deficiency. The family was lost to follow-up	Case 1. Terapia con vitamina B12. Persiste lieve ipotonia ed alterazioni MRI dopo 9 mesi. Caso 2. Vitamin B12 therapy. Hypotonia, microcephaly and speech delay persist	After six weeks, EEG was normal and cranial MRI after 10 weeks showed complete disappearance of all structural abnormalities. Cognitive and language development, however, remained seriously retarded at the age of 2 years.	Vitamin B 12 therapy, with improvement of clinical and imaging abnormalities	Case 1.<br>Vitamin B 12 therapy, the tremulous body movements lasted 6 weeks, walked at 16 months, his speech was mildly delayed<br><br>Case 2.<br>2 injections<br>of cobalamin to a total of 1.5 mg. The mother subsequently wrote to say that the child had returned to normal<br><br>Case 3.<br>Cobalamin injections were started in a dose of 0.5 mg daily. They lasted -90 s and during them the child<br>was unresponsive, the pupils were dilated, the eyes deviated<br>to the left, there was drooling, and the right armjerked.

Risk of overweight and obesity

3. Do healthy infants on a complementary feeding that is completely or partially free of any animal-based foods<br>***3.1. have a different risk of developing NCDs (Obesity/Overweight/)?***<br>***compared to infants on a balanced omnivorous diet?***	**P.** Will a healthy infant at the beginning and during the complementary feeding period (6-24 months)<br>**I.** who is offered a diet completely or partially devoid of food of animal origin<br>**C.** towards an infant who, in the same period, is offered a balanced omnivorous diet<br>**O.** have a different risk of developing NCDs (Obesity / Overweight)?

Table S3g: Included studies. Characteristics and Results

Study (First Author, year, Country/Setting)	Study design	Population (sample size, baseline characteristics)	Intervention/exposure	Primary Outcome	Measure of effects	Secondary Outcomes	Follow-up	Results	Funding
Weder et al. 2019 [43]<br>Germany	Cross-sectional study	430 children aged 1-3 years (127 vegetarian (VG), 139 vegan (VN), and 164 OM) in Germany with no diagnosed diseases that could affect the studied variables and that do not follow special diets other than vegan or vegetarian diets	A 3-day weighed dietary record assessed dietary intake, and an online questionnaire assessed lifestyle, body weight, and height.<br>.	To compare the intake of energy, macronutrients, and fiber, as well as BW and BH, of VG, VN, and OM children aged 1–3 years in Germany.	Values are unadjusted arithmetic mean ± standard deviation (SD), whereas p-values and e ect sizes were derived from ANCOVA and adjusted for age and sex (basic model) or other confounders<br><br>Weight-for-height z-scores<br>Weight-for-Age z-score<br>Height-for-age z-scores		N.A.	The results showed no significant difference: 36/266 children on a vegetarian/vegan diet were overweight or obese compared to 23/164 children on an omnivorous diet. (OR [95% CI] = 0.96 [0.55, 1.69]; p=0.89) (Supplementary file S4, Analysis 1.3)	Not declared

Vitamin or other micronutrient deficiencies

3. Do healthy infants on a complementary feeding that is completely or partially free of any animal-based foods<br>***3.2. have a different risk of developing vitamin or other micronutrient deficiency compared to infants on a balanced omnivorous diet?***	**P.** Will a healthy infant at the beginning and during the complementary feeding period (6-24 months)<br>**I.** who is offered a diet completely or partially devoid of food of animal origin<br>**C.** towards an infant who, in the same period, is offered a balanced omnivorous diet<br>**O.** have a different risk of developing vitamin or micronutrient deficiencies?

Table S3.h: Included Guidelines and other Documents: Recommendations and Grading

Guidelines – other Documents		Recommendations	Grading
Position Paper SIPPS - FIMP - SIMA – SIMP 2017 [23]	Case report<br>low quality	scientific evidence only consists of case reports or case series, essentially of infants older than 6 months breastfed by vegetarian or vegan mothers, admitted to the emergency department with several neurological symptoms, who had then received a diagnosis of well-documented micronutrient deficiencies.<br><br>It recommends close nutritional monitoring of the infant, even after the start of CF, with supplementation as needed to avoid severe clinical outcomes such as growth deficits, anaemia and especially neurological deficits	strong positive recommendation

Table S3i: Included studies. Characteristics and Results.

Study (First Author, year, Country/Setting)	Study design	Population (sample size, baseline characteristics)	Intervention/exposure	Primary Outcome	Measure of effects	Secondary Outcomes	Follow-up	Results	Funding
Dagnelie et al. 1994 [42]<br>The Netherlands	cross sectional study followed by mixed-longitudinal study3.	N 243 (0-8 years)<br>2 years later same cohort N 194<br><br>study 2.<br>mixed longitudinal cohort study:	Three cohorts of infants were monitored.<br>During this period, data on dietary intake, growth, blood chemistry, psycho-motor development, and physical deficiency	Cases of malnutrition in macrobiotic children incidental, or ubiquitous deficiencies	Longitudinal cohort study:<br>- food records were kept by the mothers<br>-<br>-Blood sampling and laboratory analyses		2 years	Energy intake, protein intake, Calcium, riboflavin, and vitamin B-12 intake was considerably lower in the macrobiotic group<br>- Dietary fiber intake in macrobiotic infants was increased<br>- Iron deficiency was observed.<br>Plasma vitamin B-12 concentrations, Hematocrit and red blood cell count	School of Psychology University of Nottingham (Within the last 5 years, ET and NP have received co-funding from Nutricia/Danone to support an ESRC CASE PhD studentship)

		3 cohorts 4-10mo, 8.14 mo, 12-18 mo white children with birth weight ≥2500 g and who did not suffer from a congenital disease. Children and their mothers should have been on a macrobiotic diet from birth of the child onward (n = 53). Omnivorous control subjects (n = 57)<br><br>study 3.<br>Intervention study: Macrobiotic infants with the most clear-cut nutritional deficiencies (n = 27 from the original sample of 53 infants)	symptoms were collected.<br><br>Intervention study: Intensive consultations with macrobiotic teachers in The Netherlands and abroad, were initiated. As a result, a joint report was handed out to all parents participating in the study<br><br>complementary feeding of macrobiotic infants started at 4. 8 moₒ. In the control group, complementary feeding started with fruits at mean age 2.7 mo					were significantly lower, whereas mean corpuscular volume and mean corpuscular hemoglobin mass were significantly higher. Mean folate concentrations were higher.<br>- Plasma riboflavin, 25-hydroxyvitamin D, calcium, and phosphate concentrations were significantly lower<br><br>Following recommendations<br>1) Add dietary fat as a source of energy to 25-30% of energy intake in children, by including 20-25 g oil/d or twice this amount of nuts and seeds.<br>2) As a source of vitamin B- I 2, products of animal origin such as fish are needed. Consume only moderate amounts of sea- weeds because these contain non bioavaiable vitamin B-l2 analogues, which may block vitamin B-12 metabolism.<br>3) Inclusion of dairy products as a source of calcium, protein, and riboflavin.	
Taylor et al. 2004 [53] Guildford, Surrey GU2 7XH, UK	longitudinal prospective study	The study group was recruited from a generally prosperous, middle- class population who were well nourished 198 infants who weighed at least 2500 gr at birth.<br><br>Parents were free to choose the type and amount of meat offered to their children.	Seven-day weighed food intake diaries were recorded when the infants were at 4, 8, 12, 16, 20, and 24 mo of age.<br><br>Mothers/carers were given careful instruction (both verbally and in writing) on the preparation/completion of weighed food intake records. The diaries were reviewed by the study nurses together with the parent/carer to identify any anomalous or potentially<br><br>Do not specify any supplementations, nor	to determine whether the iron status of infants and young children benefits from the addition of meat to the diet	At 4, 12, and 24 mo of age, capillary blood specimens (300–400 ìL) were collected. Whole-blood, and analyzed for hematological parameters of iron and micronutrient status.	to determine the impact of meat consumption on zinc and copper status in infants and young children	From 4 mo of age to 24 mo of age	At 4 mo: no differences in mean daily intakes of any nutrient among the four diet groups were seen<br><br>Differences were observed for dietary iron intake at 16, 20, and 24 mo, with intakes being higher in nonmeat eaters than the rest (*p*<0.024, *p*<0.011, and *p*<0.014, respectively)<br><br>no statistically significant differences between groups were found at any age thus there was no clear association between dietary meat consumption and iron status,<br><br>significant inverse association between the number of low serum iron results	Undeclared

			the% of children breastfed or formula-fed<br><br>Subjects were classified as nonmeat eaters or as mixed (red and white)-meat eaters subgrouped into tertiles depending on the meat content reported in diet diaries.<br><br>The program calculates the daily intake of energy and of macronutrients and micronutrients from the diet records and compares these results with the appropriate reference data<br><br>The food composition database provided with the Comp-Eat program was regularly updated by research nurses as new infant food products became commercially available.					(<9.0 ìmol/L) and intake of meat at 12 mo of age (*p*<0.023).<br><br>10% of the subjects had zinc intakes below the UK RNI of 5 mg/d at 24 mo<br><br>no statistically significant differences in serum zinc levels among the diet groups were found at any age<br><br>No statistically significant differences in serum copper levels among the diet groups were found at any age	

See Table S3f: Included Case reports. Characteristics and Results

**CASE REPORT**	Blasco-Alonso et al. [46]	Lemoine et al. [47]	Lund et al. [48]	Smolka et al. 2001 [49]	von Schenck et al. 1997 [50]	Lövblad et a.1997 [51]	Grattan-Smith et al. 1997 [52]
