# Supplementary material for: Do Vegetarian Diets Provide Adequate Nutrient Intake during Complementary Feeding? A Systematic Review
Source: Nutrients. 2022 Aug 31;14(17):3591. doi: 10.3390/nu14173591 (PMC9459879; doi:10.3390/nu14173591)

Supplementary file S4. ADDITIONAL ANALYSIS OF THE RESULTS

1. Growth and risk of overweight / obesity in the age of complementary feeding in children on a diet lacking animal protein compared to omnivorous children

Analysis 1.1. Risk of wasted growth

Study or Subgroup	macrobiotic diet Events	macrobiotic diet Total	omnivorous diet Events	omnivorous diet Total	Weight	Risk Ratio M-H, Fixed, 95% CI
Degnelie 1994	15	49	1	57	100.0%	17.45 [2.39, 127.38]
**Total (95% CI)**		**49**		**57**	**100.0%**	**17.45 [2.39, 127.38]**
Total events	15		1			
Heterogeneity: Not applicable						
Test for overall effect: Z = 2.82 (P = 0.005)						

Risk Ratio
M-H, Fixed, 95% CI

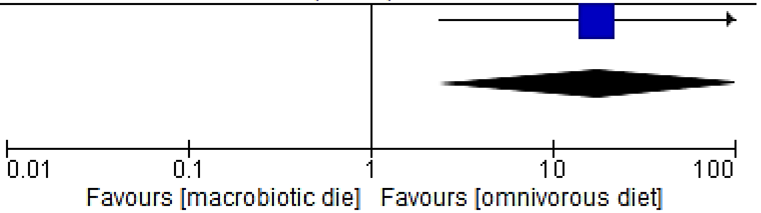

Analysis 1.2. Risk of stunted or wasted growth

Study or Subgroup	vegan/vegetarian diet Events	vegan/vegetarian diet Total	omnivorous diet Events	omnivorous diet Total	Weight	Odds Ratio M-H, Fixed, 95% CI
Weder 2019	21	266	1	164	100.0%	13.97 [1.86, 104.88]
**Total (95% CI)**		**266**		**164**	**100.0%**	**13.97 [1.86, 104.88]**
Total events	21		1			
Heterogeneity: Not applicable						
Test for overall effect: Z = 2.56 (P = 0.01)						

Odds Ratio
M-H, Fixed, 95% CI

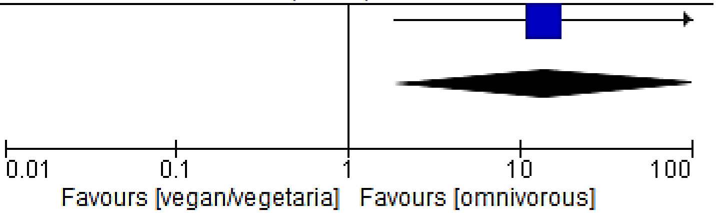

Analysis 1.3. Risk of overweight / obesity

	vegan/vegetarian diet		omnivorous diet			Odds Ratio
**Study or Subgroup**	**Events**	**Total**	**Events**	**Total**	**Weight**	**M-H, Fixed, 95% CI**
Weder 2019	36	266	23	164	100.0%	0.96 [0.55, 1.69]
**Total (95% CI)**		**266**		**164**	**100.0%**	**0.96 [0.55, 1.69]**
Total events	36		23			

Heterogeneity: Not applicable
Test for overall effect: Z = 0.14 (P = 0.89)

Odds Ratio
M-H, Fixed, 95% CI

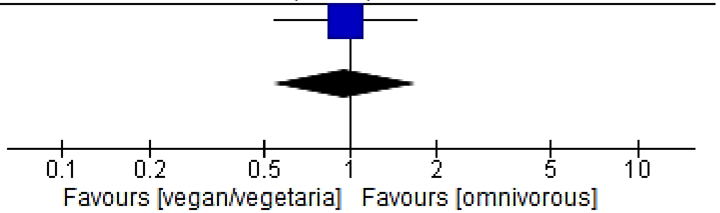

Supplement: Supplementary file 1 [file nutrients-14-03591-s001.zip › nutrients_1869505_Supplementary file S4.pdf]
