# Supplementary material for: Do Vegetarian Diets Provide Adequate Nutrient Intake during Complementary Feeding? A Systematic Review
Source: Nutrients. 2022 Aug 31;14(17):3591. doi: 10.3390/nu14173591 (PMC9459879; doi:10.3390/nu14173591)
Supplement: Supplementary file 1 [file nutrients-14-03591-s001.zip › nutrients_1869505_Supplementary file S5.pdf]

File S5. SUMMARY OF FINDINGS FOR THE MAIN COMPARISON

Table S5a: Vegetarian diet during the complementary feeding period. Different auxological development and/or growth

[complementary feeding completely or partially free of animal-source foods] compared to [balanced omnivorous diet] for [different auxological development and/or growth]

Patient or population: [different auxological development and/or growth]

Setting: Primary care

Intervention: [complementary feeding completely or partially free of animal-source foods]

Comparison: [balanced omnivorous diet]

Outcomes	№ of participants (studies) Follow-up	Certainty of the evidence (GRADE)	Relative effect (95% CI)	Anticipated absolute effects	
				Risk with [balanced omnivorous diet]	Risk difference with [complementary feeding completely or partially free of animal-source foods]
Risk of wasted growth<br>assessed with: % infant with major skin and muscle wasting<br>follow-up: 2 years	110<br>(1 observational study)<br>[42]	⊕⊕⊕◯<br>Moderate[a,b,c]	**RR 17.45**<br>(2.39 to 127.38)	18 per 1.000	**289 more per 1.000**<br>(24 more to 2.217 more)
Risk of stunted or wasted growth<br>assessed with: % children with stunted growth	430<br>(1 observational study)<br>[43]	⊕⊕⊕◯<br>Moderate[c,d]	**OR 13.97**<br>(1.86 to 104.88)	6 per 1.000	**73 more per 1.000**<br>(5 more to 385 more)

[complementary feeding completely or partially free of animal-source foods] compared to [balanced omnivorous diet] for [different auxological development and/or growth]

Patient or population: [different auxological development and/or growth]

Setting: Primary care

Intervention: [complementary feeding completely or partially free of animal-source foods]

Comparison: [balanced omnivorous diet]

Outcomes	№ of participants (studies) Follow-up	Certainty of the evidence (GRADE)	Relative effect (95% CI)	Anticipated absolute effects	
				Risk with [balanced omnivorous diet]	Risk difference with [complementary feeding completely or partially free of animal-source foods]

*__The risk in the intervention group__ (and its 95% confidence interval) is based on the assumed risk in the comparison group and the **relative effect** of the intervention (and its 95% CI).
CI: confidence interval

GRADE Working Group grades of evidence
High certainty: we are very confident that the true effect lies close to that of the estimate of the effect.
Moderate certainty: we are moderately confident in the effect estimate: the true effect is likely to be close to the estimate of the effect, but there is a possibility that it is substantially different.
Low certainty: our confidence in the effect estimate is limited: the true effect may be substantially different from the estimate of the effect.
Very low certainty: we have very little confidence in the effect estimate: the true effect is likely to be substantially different from the estimate of effect.

Explanations

a. Ascertainment of the exposure
b. a small sample population, inappropriately detailed diets, generically defined as "omnivorous" or "adequate" and "inadequate,"
c. wide 95% C.I.
d. study design cross-sectional; single study

Table S5b: Vegetarian diet during the complementary feeding period. Psychomotor development

[complementary feeding completely or partially free of animal-source foods] compared to [balanced omnivorous diet] for [psychomotor development that is significantly different]

Patient or population: [psychomotor development that is significantly different]

Setting: Primary care

Intervention: [complementary feeding completely or partially free of animal-source foods]

Comparison: [balanced omnivorous diet]

Outcomes	№ of participants (studies) Follow-up	Certainty of the evidence (GRADE)	Relative effect (95% CI)	Anticipated absolute effects	
				Risk with [balanced omnivorous diet]	Risk difference with [complementary feeding completely or partially free of animal-source foods]
Psychomotor development assessed with: standardized psychomotor checklist (score)	110 (1 observational study) [42]	⊕⊕◯◯ Low[a,b]	-	The mean psychomotor development was **0**	-0.63 **0** (0 to 0)
Psychomotor development assessed with: case report e case series	10 (7 case reports) [46-52]	⊕⊕⊕◯ Moderate[c]	10 cases of children aged 8-18 months: all of them exhibit severe neurological outcomes and growth deficits resulting from low vitamin B12 and vitamin D levels, with anaemia, stunting, brain abnormalities, and demyelination. Cases with persistent outcomes are reported with no long-term follow-up data for any other disorders.		

[complementary feeding completely or partially free of animal-source foods] compared to [balanced omnivorous diet] for [psychomotor development that is significantly different]

Patient or population: [psychomotor development that is significantly different]

Setting: Primary care

Intervention: [complementary feeding completely or partially free of animal-source foods]

Comparison: [balanced omnivorous diet]

Outcomes	№ of participants (studies) Follow-up	Certainty of the evidence (GRADE)	Relative effect (95% CI)	Anticipated absolute effects	
				Risk with [balanced omnivorous diet]	Risk difference with [complementary feeding completely or partially free of animal-source foods]

*__The risk in the intervention group__ (and its 95% confidence interval) is based on the assumed risk in the comparison group and the **relative effect** of the intervention (and its 95% CI).
CI: confidence interval

GRADE Working Group grades of evidence

High certainty: we are very confident that the true effect lies close to that of the estimate of the effect.
Moderate certainty: we are moderately confident in the effect estimate: the true effect is likely to be close to the estimate of the effect, but there is a possibility that it is substantially different.
Low certainty: our confidence in the effect estimate is limited: the true effect may be substantially different from the estimate of the effect.
Very low certainty: we have very little confidence in the effect estimate: the true effect is likely to be substantially different from the estimate of effect.

Explanations

a. single study; not detailed data report
b. unclear
c. case report and case series

Table S5c: Vegetarian diet during the complementary feeding period. Risk of developing Obesity/Overweight

[complementary feeding completely or partially free of animal-source foods] compared to [balanced omnivorous diet] for [risk of developing NCDs (Obesity/Overweight)]

Patient or population: [risk of developing NCDs (Obesity/Overweight)]

Setting: Primary care

Intervention: [complementary feeding completely or partially free of animal-source foods]

Comparison: [balanced omnivorous diet]

Outcomes	№ of participants (studies) Follow-up	Certainty of the evidence (GRADE)	Relative effect (95% CI)	Anticipated absolute effects	
				Risk with [balanced omnivorous diet]	Risk difference with [complementary feeding completely or partially free of animal-source foods]
Risk of overweight / obesity assessed with: % children with overweight / obesity	430 (1 observational study) [43]	⨁⨁⨁◯ Moderate[a]	**OR 0.96** (0.55 to 1.69)	140 per 1.000	**5 fewer per 1.000** (58 fewer to 76 more)

*__The risk in the intervention group__ (and its 95% confidence interval) is based on the assumed risk in the comparison group and the **relative effect** of the intervention (and its 95% CI).

CI: confidence interval; **OR:** odds ratio

[complementary feeding completely or partially free of animal-source foods] compared to [balanced omnivorous diet] for [risk of developing NCDs (Obesity/Overweight)]

Patient or population: [risk of developing NCDs (Obesity/Overweight)]

Setting: Primary care

Intervention: [complementary feeding completely or partially free of animal-source foods]

Comparison: [balanced omnivorous diet]

Outcomes	№ of participants (studies) Follow-up	Certainty of the evidence (GRADE)	Relative effect (95% CI)	Anticipated absolute effects	
				Risk with [balanced omnivorous diet]	Risk difference with [complementary feeding completely or partially free of animal-source foods]

GRADE Working Group grades of evidence

High certainty: we are very confident that the true effect lies close to that of the estimate of the effect.
Moderate certainty: we are moderately confident in the effect estimate: the true effect is likely to be close to the estimate of the effect, but there is a possibility that it is substantially different.
Low certainty: our confidence in the effect estimate is limited: the true effect may be substantially different from the estimate of the effect.
Very low certainty: we have very little confidence in the effect estimate: the true effect is likely to be substantially different from the estimate of effect.

Explanations

a. study design cross-sectional; single study

Table S5d: Vegetarian diet during the complementary feeding period. Risk of developing vitamin or other micronutrient deficiencies

[complementary feeding completely or partially free of animal-source foods] compared to [balanced omnivorous diet] for [risk of developing vitamin or other micronutrient deficiencies]

Patient or population: [risk of developing vitamin or other micronutrient deficiencies]

Setting: Primary care

Intervention: [complementary feeding completely or partially free of animal-source foods]

Comparison: [balanced omnivorous diet]

Outcomes	№ of participants (studies) Follow-up	Certainty of the evidence (GRADE)	Anticipated absolute effects	
			Risk with [balanced omnivorous diet]	Risk difference with [complementary feeding completely or partially free of animal-source foods]
Risk of vitamin or other micronutrient deficiencies follow-up: 24 months	308<br>(2 observational studies) [42, 53]	⊕⊕◯◯ Low[a,b]	Calcium, riboflavin, and vitamin B-12 intake was considerably lower in the macrobiotic group<br>- Dietary fiber intake in macrobiotic infants was increased<br>- Iron deficiency was observed. Plasma vitamin B-12 concentrations, Hematocrit and red blood cell count were significantly lower, whereas mean corpuscular volume and mean corpuscular hemoglobin mass were significantly higher. Mean folate concentrations were higher.<br>- Plasma riboflavin, 25-hydroxyvitamin D, calcium, and phosphate concentrations were significantly lower<br>Differences were observed for dietary iron intake at 16, 20, and 24 mo, with intakes being higher in nonmeat eaters than the rest (p<0.024, p<0.011, and p<0.014, respectively)	

[complementary feeding completely or partially free of animal-source foods] compared to [balanced omnivorous diet] for [risk of developing vitamin or other micronutrient deficiencies]

Patient or population: [risk of developing vitamin or other micronutrient deficiencies]

Setting: Primary care

Intervention: [complementary feeding completely or partially free of animal-source foods]

Comparison: [balanced omnivorous diet]

Outcomes	№ of participants (studies) Follow-up	Certainty of the evidence (GRADE)	Anticipated absolute effects	
			Risk with [balanced omnivorous diet]	Risk difference with [complementary feeding completely or partially free of animal-source foods]
Risk of vitamin or other micronutrient deficiencies assessed with: case report e case series	10<br>(7 case reports) [46-52]	⊕⊕◯◯ Low[c]		

*__The risk in the intervention group__ (and its 95% confidence interval) is based on the assumed risk in the comparison group and the **relative effect** of the intervention (and its 95% CI).

CI: confidence interval

GRADE Working Group grades of evidence
High certainty: we are very confident that the true effect lies close to that of the estimate of the effect.
Moderate certainty: we are moderately confident in the effect estimate: the true effect is likely to be close to the estimate of the effect, but there is a possibility that it is substantially different.
Low certainty: our confidence in the effect estimate is limited: the true effect may be substantially different from the estimate of the effect.
Very low certainty: we have very little confidence in the effect estimate: the true effect is likely to be substantially different from the estimate of effect.

Explanations

a. Taylor et al. does not specify whether the vegetarian group ate foods supplemented with iron. In Dagnelie et al. small sample population, inappropriately detailed diets, generically defined as "omnivorous" or "adequate" and "inadequate,"
b. The two studies have conflicting results (methodological biases)
c. case report and case series
